# A Candidate Gene Approach Identifies the *CHRNA5-A3-B4* Region as a Risk Factor for Age-Dependent Nicotine Addiction

**DOI:** 10.1371/journal.pgen.1000125

**Published:** 2008-07-11

**Authors:** Robert B. Weiss, Timothy B. Baker, Dale S. Cannon, Andrew von Niederhausern, Diane M. Dunn, Nori Matsunami, Nanda A. Singh, Lisa Baird, Hilary Coon, William M. McMahon, Megan E. Piper, Michael C. Fiore, Mary Beth Scholand, John E. Connett, Richard E. Kanner, Lorise C. Gahring, Scott W. Rogers, John R. Hoidal, Mark F. Leppert

**Affiliations:** 1 Department of Human Genetics, Eccles Institute of Human Genetics, University of Utah School of Medicine, Salt Lake City, Utah, United States of America; 2 Department of Medicine, Center for Tobacco Research and Intervention, University of Wisconsin School of Medicine, Madison, Wisconsin, United States of America; 3 Department of Psychiatry, University of Utah School of Medicine, Salt Lake City, Utah, United States of America; 4 Division of Biostatistics, School of Public Health, University of Minnesota, Minneapolis, Minnesota, United States of America; 5 Department of Internal Medicine, Division of Respiratory, Critical Care and Occupational Pulmonary Medicine, University of Utah School of Medicine, Salt Lake City, Utah, United States of America; 6 Geriatric Research Education and Clinical Center, Salt Lake City VA Medical Center, Salt Lake City, Utah, United States of America; 7 Department of Neurobiology and Anatomy, University of Utah School of Medicine, Salt Lake City, Utah, United States of America; The Wellcome Trust Centre for Human Genetics, University of Oxford, United Kingdom

## Abstract

People who begin daily smoking at an early age are at greater risk of long-term nicotine addiction. We tested the hypothesis that associations between nicotinic acetylcholine receptor (nAChR) genetic variants and nicotine dependence assessed in adulthood will be stronger among smokers who began daily nicotine exposure during adolescence. We compared nicotine addiction—measured by the Fagerstrom Test of Nicotine Dependence—in three cohorts of long-term smokers recruited in Utah, Wisconsin, and by the NHLBI Lung Health Study, using a candidate-gene approach with the neuronal nAChR subunit genes. This SNP panel included common coding variants and haplotypes detected in eight α and three β nAChR subunit genes found in European American populations. In the 2,827 long-term smokers examined, common susceptibility and protective haplotypes at the *CHRNA5-A3-B4* locus were associated with nicotine dependence severity (*p* = 2.0×10^−5^; odds ratio = 1.82; 95% confidence interval 1.39–2.39) in subjects who began daily smoking at or before the age of 16, an exposure period that results in a more severe form of adult nicotine dependence. A substantial shift in susceptibility versus protective diplotype frequency (AA versus BC = 17%, AA versus CC = 27%) was observed in the group that began smoking by age 16. This genetic effect was not observed in subjects who began daily nicotine use after the age of 16. These results establish a strong mechanistic link among early nicotine exposure, common *CHRNA5-A3-B4* haplotypes, and adult nicotine addiction in three independent populations of European origins. The identification of an age-dependent susceptibility haplotype reinforces the importance of preventing early exposure to tobacco through public health policies.

## Author Summary

Tobacco use is a global health care problem, and persistent smoking takes an enormous toll on individual health. The onset of daily smoking in adolescence is related to chronic use and severe nicotine dependence in adulthood. Since nicotine is the key addictive chemical in tobacco, we tested the hypothesis that genetic variants within nicotinic acetylcholine receptors will influence the severity of addiction measured in adulthood. Using genomic resequencing to define the patterns of variation found in these candidate genes, we observed that common haplotypes in the *CHRNA5-A3-B4* gene cluster are associated with adult nicotine addiction, specifically among those who began daily smoking before age 17. We show that in populations of European origins, one haplotype is a risk factor for dependence, one is protective, and one is neutral. These observations suggest that genetic determinants expressed during human adolescence contribute to the risk of lifetime addiction severity produced from early onset of cigarette use. Because disease risk from the adverse health effects of tobacco smoke is related to lifetime tobacco exposure, the finding that an age-dependent effect of these haplotypes has a strong influence on lifetime smoking behavior reinforces the public health significance of delaying smoking onset.

## Introduction

Nicotine addiction has profound clinical and public health consequences because it is associated with reduced ability to cease tobacco use [1],[2], and tobacco use is the leading cause of preventable morbidity and mortality in developed countries [3]. Meta-analysis of numerous twin studies shows that both genes and environment play an important role in smoking-related behaviors [4]. Nicotine is the primary agent in tobacco smoke that leads to addiction, and while progress has been made in finding genes that contribute to nicotine addiction in humans [5]–[9], there is a great need for additional progress.

Both human and animal research support an *a priori* hypothesis that age of onset of daily smoking may influence the association of genetic risk variants with measures of nicotine dependence. In humans, early onset of smoking is associated with greater consumption of cigarettes in adulthood [10]–[15], a relative inability to quit smoking [10], [16]–[18], and a more severe form of nicotine dependence [13], [19]–[22]. Research in rodents shows that nicotine exposure during periadolescence induces long-lasting biochemical, anatomical, physiological, and behavioral changes that differ markedly from those seen with adult exposure [23]–[27].

The neuronal nicotinic acetylcholine receptor genes (nAChRs) are likely candidates for harboring functional variants contributing to nicotine addiction since these ligand-gated ion channels are the initial physiological targets of nicotine in the central and peripheral nervous system. They have also been implicated in nicotine addiction in animals where chronic nicotine exposure leads to persistent changes in brain nAChRs [28],[29], and where engineered mouse models support the crucial role of the α4β2 nAChRs in nicotine addiction [30],[31]. Previous candidate gene association studies using six *CHRNA4* SNPs found evidence for associations with measures of nicotine dependence in Chinese men [5], and females of European-American and African-American descent [6]. A wider survey of nAChR gene variants in a case-control study for nicotine dependence found evidence for nominally significant associations in *CHRNA7*, *CHRNA9*, *CHRNA5* and *CHRNB3* in young Israeli women [7]. Several recent genome-wide association studies (GWAS) using either nicotine dependent smokers as cases and non-dependent smokers as controls [8] or cigarettes per day as a quantitative trait [32] have failed to yield statistically significant findings at the genome-wide level. However, when these studies independently examined nAChR candidate genes [9],[32], evidence was found for associations between common variants in *CHRNB3* and the *CHRNA5-A3-B4* gene cluster at 15q 24 and their respective phenotypes. Most recently, and subsequent to submission of this article, three separate GWAS reports provide strong evidence for an association between SNP variation at 15q24 and lung cancer [33]–[35]. One study suggests that the effect of 15q24 variants on lung cancer is primarily mediated through smoking behavior [35], while the other studies failed to associate 15q24 variants with smoking behavior and suggest that the disease mechanism with lung cancer is not explained by an association with nicotine addiction [33],[34].

This report describes results of a comprehensive haplotype discovery and nicotine addiction association study within nAChRs genes across three European American populations of 2,827 long-term smokers. Moreover, it tests the *a priori* hypothesis that associations between nAChR genetic variants and nicotine dependence severity assessed in adulthood will be stronger among smokers who began daily smoking in adolescence than among those who did not. The results show significant dependence-haplotype associations in the *CHRNA5-A3-B4* gene cluster occurring only in the early onset subjects, consistent with the hypothesis that the association of genetic risk variants for nicotine addiction may be influenced by the age of onset of daily smoking.

## Results

### Study Populations

Nicotine dependence was assessed with the Fagerstrom Test of Nicotine Dependence (FTND) because it predicts important dependence attributes such as the likelihood of relapse back to tobacco use and biochemical measures of nicotine self-administration [2],[36],[37]. The FTND assesses a pattern of heavy, compulsive smoking and has been used in other genetic association studies [5]–[9]. Genetic variants were assessed in 2,827 subjects from three European American cohorts with a mean age of 49.6 years (SD = 9.5), 1155 (41%) of whom were females (Table S1). All participants were either current or previous daily cigarette smokers; 222 (8%) had not smoked for at least 2 years prior to participation in the study. One cohort comprised participants in a study of genetic risk factors for nicotine dependence and chronic obstructive pulmonary disease (COPD) recruited in Salt Lake City, Utah (N = 486, UT). Another cohort was made up of participants in randomized trials of smoking cessation interventions recruited in Madison and Milwaukee, Wisconsin (N = 398, WI). A final cohort was drawn from the Lung Health Study (N = 1943, LHS), a multi-site longitudinal study of COPD sponsored by the Division of Lung Disease of the National Heart, Lung and Blood Institute [38]. All ex-smokers were in the UT cohort and responded to smoking related assessments based upon their prior smoking patterns [39]. Participants began daily smoking at a mean age of 17.3 (SD = 4.1), smoked a mean of 28.3 cigarettes per day (CPD, SD = 13.9), smoked for a mean 30.7 years (SD = 9.5), and had a mean FTND score of 5.7 (SD = 2.2). Consistent with a history of chronic, heavy smoking, most subjects in the UT and LHS cohorts (N = 2302, 81% of the total sample) had mild to moderate chronic obstructive pulmonary disease (COPD) as determined by pulmonary function testing; lung function testing was not performed on the WI subjects.

Age of onset of daily smoking was dichotomized into early onset (onset of daily smoking at age 16 or younger) vs. late onset (onset of daily smoking at age 17 or older). Previous studies have shown that 16 vs. 17 is an appropriate age range to differentiate the impact of early from late nicotine exposure on dependence [40]. We used a dichotomous variable to represent age because the effect was hypothesized to be nonlinear in nature due to the fact that nicotine exerts distinct effects when administered during adolescence vs. adulthood [24]. We attempted to reflect this underlying causal model in our analyses by creating an age cut-score that reflected this nonlinear effect. Also, the age 16 vs. 17 dichotomy approximated a median split, which yielded near equivalent statistical power for tests within the two age samples; 46% of the sample were thus classified as early onset smokers. Research suggests that age of smoking onset can be reliably assessed with retrospective assessments [41]. To compare distinct levels of nicotine dependence, FTND scores were dichotomized into low (FTND = 0–4) and high (FTND = 6–10) dependence [7]. An FTND score of 6 or higher identifies subjects with high nicotine dependence [42], while a score of “5” has an ambiguous relation with dependence [42]–[44]. Of the initial sample of 2827, 731 (26%) were assigned to the low dependence condition, 1556 (55%) were assigned to the high dependence condition and 540 (19%) had an intermediate score of 5.

Consistent with previous reports, age of onset of daily smoking was inversely related to level of dependence in the present sample. Age of onset and FTND score were correlated, r(2135) = −0.18, *P*<0.001, and dichotomized age of onset and FTND were highly associated (χ2 = 51.6, 1 df, *P*<0.001). In early onset smokers, 24.2% had low FTND scores compared with 38.7% of late onset smokers.

### SNP and Haplotype Discovery

As an initial step, we conducted an exhaustive SNP discovery survey surrounding the neuronal nAChR coding regions in a small sample selected to represent the most extreme heavy and light dependent smokers. We used a genomic resequencing strategy to generate a dense set of variants in α-like nAChR subunits (α2, α3, α4, α5, α6, α9, and α10) and β-like nAChR subunits (β2, β3, and β4) in 144 smokers and 48 population-matched non-smokers. This survey identified 262 SNPs, including 38 nonsynonymous, 35 synonymous, 57 UTR, 1 stop, and 1 frameshift (see Table S2 for location and allele frequency). The stop and frameshift alleles, both located in *CHRNA6*, were each observed as heterozygotes in single individuals, indicating that the depth of the resequencing survey reached the boundary of rare loss-of-function alleles. For a preliminary χ2 analysis of association in the resequencing sample, we subdivided the 144 smokers into high (n = 72) and low-dependent (n = 72) categories. Even with this small selected sample set, a nominally significant association signal (*P*<0.01) was observed for five SNPs located in the *CHRNA5-A3-B4* cluster on chromosome 15q25: rs951266 (intronic A5), rs16969968 (nonsynonymous A5), rs8192482 (3′-UTR A5), rs4887067 (intergenic A5-A3) and rs17487223 (intronic B4). All five SNPs are in strong linkage disequilibrium. In order to extend this survey, we defined haplotype structures in all nAChR subunits based on inference from the unphased resequencing data, and derived a minimal set of tagging SNPs (n = 87) for genotyping in the larger three-cohort sample.

**Figure 1:**
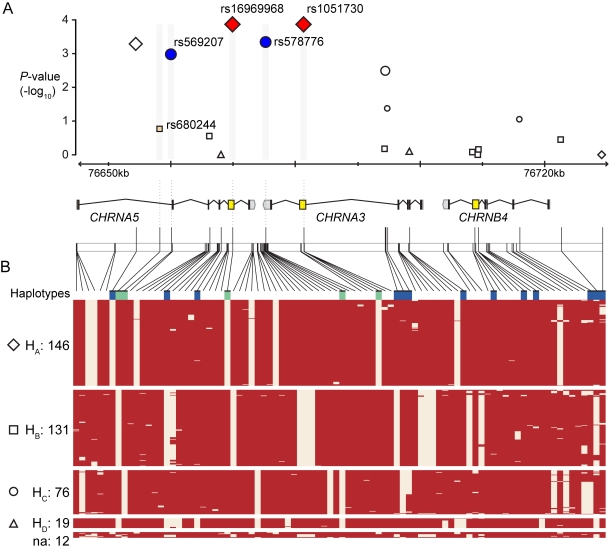
Haplotype Structure and Association Results in the Nicotinic α5, α3 and β4 Receptor Subunit Genes on Chromosome 15q24. (A) Genomic region of *CHRNA5-A3-B4* transcription units on chr. 15 between 76,644,000 to 76,732,000 base pairs (NCBI Build 36). −log_10_(P value) plot of SNPs as a function of genomic position, with their P values (allele association values from chi square tests) observed in the early onset condition. The symbols indicate haplotype assignment (H_A_, H_B_, H_C_ and H_D_) of the individual markers; open symbols indicate P values in the UT-WI cohorts and shaded symbols indicate P values in the combined UT-WI-LHS cohorts. The five SNPs used to assign haplotype status in the UT-WI-LHS cohorts are colored by haplotype affiliation: red (H_A_), (blue (H_C_) or beige (H_B_). (B) fastPHASE inferred haplotype structure of the region from unphased resequencing and genotypic data from 384 chromosomes (resequencing sample cohort). Haplotypes were assigned to groups using the 5 SNPs genotyped in the UT-WI-LHS cohorts (rs680244, rs569207, rs16969968, rs578776, and rs1051730). Haplotype counts for H_A_, H_B_, H_C,_ H_D_ and not assigned (na) are shown on the left, and the positions of SNPs genotyped in the UT-WI cohorts are indicated above the haplotypes by blue and in the UT-WI-LHS cohorts by green indicators.

Genotyping of all 87 tagging SNPS across the neuronal nAChR α and β subunits was first carried out in the UT (n = 439) and WI cohorts (n = 339), and single marker allelic tests using χ^2^ statistics were evaluated for association with FTND scores in early versus late onset samples (Table S3). The strongest association signals (*P*<0.005) to dichotomized FTND by age of onset were observed only in the early onset sample for six SNPs within the *CHRNA5-A3-B4* gene cluster with significant allele test *P* values ranging from 4.8×10^−3^ to 5.0×10^−4^ (Table 1). No other nAChR SNP had a *P* value less than 0.02 in either the early or late onset group. These six *CHRNA5-A3-B4* SNPs stratified into two groups of significant linkage disequilibrium (LD), and examination of phased resequencing haplotypes (Figure 1) revealed that these association signals occur within a ∼50 kb LD block-like structure spanning *CHRNA5* and *CHRNA3*. This LD block in the resequencing samples is composed of four major haplotypes: Haplotype A (H_A_) = 38%, Haplotype B (H_B_) = 34%, Haplotype C (H_C_) = 20%, Haplotype D (H_D_) = 5%.

**Table 1:** Association of *CHRNA5 – A3 – B4* SNPs with Dichotomized FTND Scores in the UT and WI Cohorts.

SNP	Gene	Location	Chr. 15 Position^a^	Minor allele	Early onset of daily smoking by 16			Late onset of daily smoking after 16		
					Frequency: Low, High^b^	*p*-Value	Allelic OR (95% CI)	Frequency: Low, High^c^	*p*-Value	Allelic OR (95% CI)
rs17486278	CHRNA5	intron 1	76654537	C	0.279, 0.417	**0.0005**	1.85 (1.31–2.62)	0.403, 0.395	0.81	0.96 (0.72–1.29)
rs680244	CHRNA5	intron 1	76658343	A	0.461, 0.419	0.32	0.84 (0.61–1.17)	0.406, 0.401	0.88	0.98 (0.73–1.31)
rs569207	CHRNA5	intron 1	76660174	A	0.260, 0.167	**0.0042**	0.57 (0.39–0.84)	0.191, 0.203	0.68	1.08 (0.75–1.54)
rs555018	CHRNA5	intron 3	76666297	C	0.462, 0.415	0.25	0.83 (0.60–1.15)	0.407, 0.400	0.85	0.97 (0.73–1.30)
rs16969968	CHRNA5	Asn348Asp	76669980	A	0.284, 0.415	**0.0009**	1.79 (1.27–2.54)	0.401, 0.400	0.97	1.00 (0.75–1.33)
rs578776	CHRNA3	3′ UTR	76675455	T	0.317, 0.218	**0.0048**	0.60 (0.42–0.86)	0.218, 0.223	0.87	1.03 (0.73–1.45)
rs1051730	CHRNA3	Gly394Gly	76681394	T	0.284, 0.415	**0.0009**	1.79 (1.27–2.54)	0.398, 0.401	0.93	1.01 (0.76–1.35)
rs2869546	CHRNA3	intron 4	76694400	C	0.394, 0.374	0.61	0.92 (0.66–1.28)	0.384, 0.374	0.77	0.96 (0.72–1.28)
rs7177514	CHRNA3	intron 4	76694461	G	0.341, 0.235	**0.0032**	0.59 (0.42–0.84)	0.229, 0.245	0.59	1.10 (0.78–1.53)
rs12443170	CHRNA3	intron 4	76694791	A	0.149, 0.096	0.038	0.61 (0.38–0.98)	0.111, 0.148	0.13	1.39 (0.91–2.13)
rs11636605	CHRNB4	intron 1	76715933	A	0.212, 0.156	0.072	0.69 (0.46–1.04)	0.159, 0.182	0.40	1.18 (0.80–1.72)
rs11633223	CHRNB4	5′ flank	76722531	C	0.414, 0.378	0.37	0.86 (0.62–1.20)	0.384, 0.376	0.81	0.96 (0.72–1.29)
rs3971872	CHRNB4	5′ flank	76729090	T	0.096, 0.092	0.86	0.95 (0.55–1.65)	0.068, 0.079	0.57	1.17 (0.68–2.02)

Data in bold indicate significant SNPs.^a^Chromosome 15 coordinates from NCBI Build 36.1 (hg18).

b Minor allele frequency in Low (FTND = 0–4, n = 106) and High (FTND = 6–10, n = 271).

c Minor allele frequency in Low (FTND = 0–4, n = 183) and High (FTND = 6–10, n = 217).

### *CHRNA5-A3-B4* Haplotype Effects

To evaluate the significance of these *CHRNA5-CHRNA3* single marker associations and the underlying haplotypes, five tagging SNPs capable of distinguishing these four haplotypes were assessed for association to FTND by age of daily smoking onset (daily smoking onset by vs. after age 16) in the combined UT-WI-LHS cohorts (total N = 2,827). Observed frequencies of Haplotypes A–D, respectively, by cohort were as follows: LHS, 39%, 37%, 19%, and 5%; UT, 38%, 38%, 20%, and 4%; and WI, 41%, 38%, 17%, and 4%. A test of all four haplotypes showed a significant omnibus association *P* value of 2.6×10^−4^ with high vs. low FTND score within the early onset group (Table 2), but not within the late onset group (*P* = 0.444). This omnibus haplotype test was supported by single marker association *P* values ranging from 1.1×10^−3^ to 1.7×10^−4^ (Figure 1). The main haplotype effect can be partitioned into two significant haplotype-specific associations (Table 2) with both a susceptibility effect for high dependence in the early onset group for haplotype H_A_ (odds ratio (OR) 1.50, 95% CI 1.21 to 1.86, *P* = 1.3×10^−4^), and a protective effect for haplotype H_C_ (OR 0.66, 95% CI 0.52 to 0.85, *P* = 1.1×10^−3^). Importantly, haplotype H_B_ shows no significant association (OR 0.93, 95% CI 0.76 to 1.14, *P* = 0.50), suggesting that the two haplotype-specific associations are the result of distinct susceptibility (H_A_) and protective (H_C_) haplotype effects in the early onset group.

**Table 2:** *CHRNA5-A3-B4* Haplotype-Specific Association with Dichotomized FTND Scores in the UT-WI-LHS Cohorts.

Haplotype	Haplotype Tags^a^	Early onset of daily smoking by 16				Late onset of daily smoking after 16			
		Frequency FTND = 0–4	Frequency FTND = 6–10	*p*_HS_	OR_HS_ (95% CI)	Frequency FTND = 0–4	Frequency FTND = 6–10	*p*_HS_	OR_HS_ (95% CI)
		(n = 256)	(n = 795)			(n = 493)	(n = 776)		
**H_A_**	CCAGA	0.318	0.413	**0.00013**	1.50 (1.21–1.86)	0.381	0.412	0.12	1.14 (0.97–1.34)
**H_B_**	TCGGG	0.375	0.358	0.50	0.93 (0.76–1.14)	0.385	0.365	0.30	0.91 (0.77–1.08)
**H_C_**	CTGAG	0.244	0.179	**0.00107**	0.66 (0.52–0.85)	0.192	0.187	0.74	0.96 (0.78–1.19)
**H_D_**	TCGAG	0.063	0.051	0.33	0.81 (0.53–1.23)	0.042	0.035	0.47	0.86 (0.57–1.29)

a The haplotype-tagging SNPs are rs680244, rs569207, rs16969968, rs578776, rs1051730, and the tag sequences are from the chr. 15 (+) strand.

Data in bold indicate significant haplotype-specific tests (*p*_hs_); an omnibus haplotype test was significant in the early onset group (*p* = 0.00026) but not in the late onset group (*p* = 0.444).

An interaction between haplotype status and age of onset in predicting dependence severity was also obtained via logistic regression analyses (Table 3). A significant interaction effect (*P* = 0.006) was found, as well as a significant H_A_ vs. H_C_ contrast within the early onset condition (*P* = 2.0×10^−5^, OR = 1.82, 95% CI 1.39 to 2.39). H_C_ was associated with reduced likelihood of severe nicotine dependence relative to H_A_, but only among those smoking daily at age 16 or younger. Gender was associated with age of onset in our cohorts (41% of females began daily smoking before age 17 compared with 49% of males). However, gender did not moderate the relation between dependence and haplotype status within age of onset groups and so was dropped from subsequent analyses. Based on these results for the entire sample, haplotype associations with the dichotomized FTND variable were tested by age of onset condition within each cohort separately (Table 4). There was a significant H_A_ vs. H_C_ contrast in the early onset group for each cohort (*P*'s = 0.02−0.001, OR's = 1.5–3.0), but this contrast was not significant in any cohort in the late onset condition. Thus, the relative risk/protective effect of haplotypes A and C was found only in early onset smokers in three separate cohorts.

**Table 3:** Logistic Regression Analyses in which Low and High Nicotine Dependence (FTND = 0–4 vs. FTND = 6–10) Was the Dependent Variable and Haplotype C Was the Reference Condition.

Haplotype	Interaction^a^			Early onset of daily smoking by 16			Late onset of daily smoking after 16		
	N	*P*	OR (95% CI)	N	*p*	OR (95% CI)	N	*p*	OR (95% CI)
**H_A_**	2210	0.006	1.65 (1.16–2.35)	988	2.0×10^−5^	1.82 (1.39–2.39)	1222	0.38	1.11 (0.88–1.39)
**H_B_**	2094	0.08	1.36 (0.96–1.94)	935	0.04	1.33 (1.02–1.74)	1159	0.83	0.98 (0.78–1.22)

a Interaction = interaction between haplotype and early vs. late onset of daily smoking.

N = haplotype count and OR = odds ratio (95% confidence intervals in parentheses). Haplotype C counts by test are: Interaction, 1089; Early Onset, 511; and Late Onset, 578.

**Table 4:** Logistic Regression Analyses by Cohort in which Low and High Nicotine Dependence (FTND = 0–4 vs. FTND = 6–10) Was the Dependent Variable and Haplotype C Was the Reference Condition.

Cohort	Haplotype	Early onset of daily smoking by 16		Late onset of daily smoking after 16	
		*p*	OR (95% CI)	*p*	OR (95% CI)
**LHS**	**H_A_**	0.02	1.52 (1.07–2.14)	0.19	1.20 (0.91–1.58)
	**H_B_**	0.21	1.25 (0.88–1.76)	0.87	0.98 (0.74–1.29)
**UT**	**H_A_**	0.001	2.97 (1.53–5.76)	0.72	0.91 (0.56–1.48)
	**H_B_**	0.27	1.42 (0.76–2.66)	0.88	0.96 (0.59–1.57)
**WI**	**H_A_**	0.02	2.16 (1.16–4.00)	0.98	1.01 (0.49–2.08)
	**H_B_**	0.13	1.58 (0.88–2.85)	0.94	1.03 (0.49–2.15)

**Figure 2:**
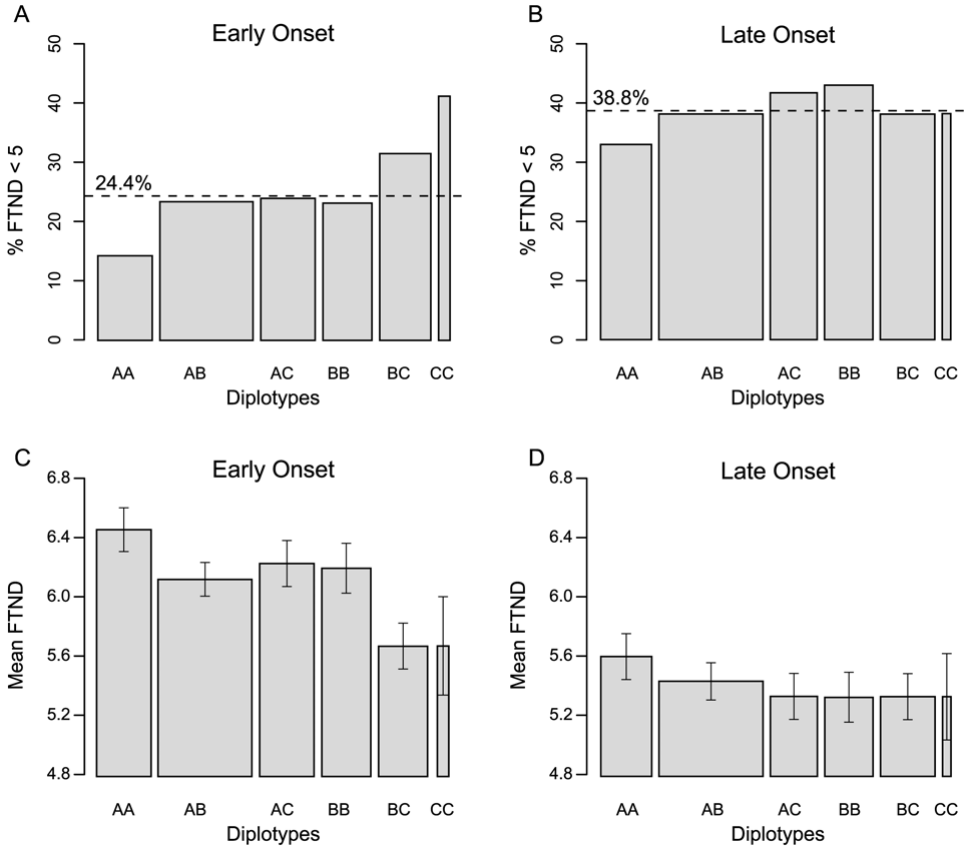
Low Nicotine Dependence (%) and Mean FTND Scores by Diplotype and Age of Onset of Daily Smoking in the Combined UT-WI-LHS Cohorts. (A), (B) partitioning of diplotypes between FTND<5 and FTND>5 categories in early and late onset groups. The percentage of individuals in the FTND<5 category is shown by the dichotomous ‘FTND cut’ value (dashed line). (C), (D) mean FTND score of diplotypes by early and late onset; error bars indicate the S.E.M. Sample size by diplotype within the early onset condition was: AA, 161; AB, 276; AC, 162; BB, 146; BC, 153; and CC, 34. Within the late onset condition, diplotype sample sizes were: AA, 203; AB, 384; AC, 186; BB, 160; BC, 190; and CC, 34. Diplotype counts are indicated by the width of each associated column within the plots.

Diplotype analyses also suggest that age-of-onset moderates the relation of these variants with nicotine dependence (Figure 2). Logistic regression analysis in the combined sample showed a significant interaction between the diplotype status (AA vs. BC) and age-of-onset (*P* = 0.02, OR = 0.43, 95% CI 0.86 to 0.21). Analyses in the early onset group found significant differences between diplotype AA and all other tested diplotypes (*P* value range from 0.05 to 3.0×10^−4^). The contrast between diplotype AA vs. diplotype AC (*P* = 0.03, OR = 0.53, 95% CI 0.93 to 0.30) and diplotype AA vs. diplotype BC (*P* = 3.0×10^−4^, OR = 0.35, 95% CI 0.62 to 0.20) suggests a recessive effect of Haplotype A, and an incomplete dominance effect of Haplotype C (Figure 2A).

**Figure 3:**
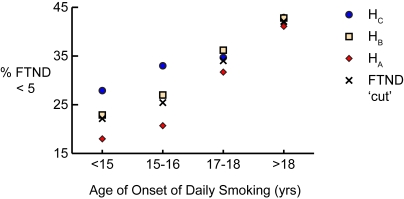
Low Nicotine Dependence (%) as a Function of Haplotypes A and C versus Age of Onset of Daily Smoking Quartiles in the UT-WI-LHS Cohorts. (A) Percentage of each haplotype in the FTND score<5 category as a function of age of onset quartiles. The percentage of individuals in the FTND<5 category for each quartile is shown by the dichotomous ‘FTND cut’ value. The age range for each quartile, with percentage of total subjects in parentheses, is as follows: <15 years (19%), 15–16 years (27%), 17–18 years (26%), and >18 years (29%).

To ensure that the obtained results were not dependent upon the particular age of daily smoking onset or FTND dichotomy that was used, the effects for both phenotypes were investigated in more detail. First, the interaction between haplotype and age of onset as a continuous variable was tested based on the combined UT-WI-LHS cohorts and using FTND46 as the dependent variable. The H_A_ vs. H_C_ by daily age interaction effect was significant, *p* = 0.01, OR = 0.95 (95% CI = 0.91–0.99), but the H_B_ vs. H_C_ interaction effect was not significant, *p* = 0.24, OR = 0.98 (95% CI = 0.94–1.02). Next, subjects where divided into quartiles based on onset age of daily smoking and the haplotype effects within each quartile were tested separately. Table 5 shows the logistic regression analysis for the H_A_ vs. H_C_ effect was significant in the two younger quartiles (*p* = 0.0003 for 15–16 years and *p* = 0.01 for <15 years), but not in the two older quartiles (*p* = 0.43 for 17–18 years and *p* = 0.63 for >18 years). Figure 3 shows the relative haplotype frequencies across each age group quartile.

**Table 5:** Logistic Regression Analysis for the H_A_ vs. H_C_ Effect by Age of Daily Smoking Onset Quartile.

Daily Age^a^	*P*-value	Odds Ratio	Upper CI	Lower CI
<15 years	0.01	1.76	2.75	1.13
15–16 years	0.0003	1.89	2.68	1.34
17–18 years	0.43	1.15	1.60	0.82
>18 years	0.63	1.08	1.46	0.79

a The age of daily smoking onset for each quartile, with percentage of total subjects in parentheses, is as follows: <15 years (19%), 15–16 years (27%), 17–18 years (26%), and >18 years (29%).

Prior research suggested that the FTND score of “5” constitutes an intermediate score that is ambiguous with regards to dependence level [42]–[44]. To explore this assumption we conducted a multinomial logistic regression with all subjects in which haplotype status was related to an FTND dependent variable that was split into three levels: “Low” (i.e., scores 0–4, N = 731), “Intermediate” (score 5, N = 540), and “High” (score>5, 1556). This analysis showed significant haplotype effects only when individuals with Low vs. High FTND scores were contrasted with one another. That is, comparisons involving individuals with Intermediate scores, yielded no significant main effects or interaction effects. An inspection of haplotype distributions showed that individuals with mid-range levels of dependence (FTND of “5”) had haplotype distributions that were intermediate to those of subjects with Low and High scores. Therefore, as would be expected if haplotypes confer dependence vulnerability, when dependence levels became more extreme, so were the relative distributions of haplotypes.

However, we also report secondary analyses with individuals with the full range of FTND scores, and with different score cut-points (i.e., FTND scores 0–4 vs. 5–10 and 0–5 vs. 6–10), in order to demonstrate that the reported pattern of results does not depend upon exclusion of mid-range scores. With both divisions (FTND scores 0–4 vs. 5–10 and 0–5 vs. 6–10), the Haplotype A vs. C interaction with age of onset was significant, as was the Haplotype A vs. C contrast within early onset but not late onset smokers (Table S4). Further, diplotype analyses indicated a significant AA vs. BC interaction with age of onset for both of the additional FTND divisions and a significant AA vs. BC contrast within the early but not the late onset condition. Inspection of the relation between FTND deciles and the frequency of H_A_ relative to H_C_ in early onset smokers indicated nonlinearity, supporting the choice to relate genetic variants to dichotomous rather than continuous FTND scores. Finally, AA vs. BC diplotypes differed significantly for mean FTND scores in early (*P* = 5×10^−4^), but not late, onset smokers (Figure 2C and D).

The psychometric performance of the six-item FTND to assess physical dependence on tobacco smoking is well established, particularly items 1 and 4 which relate to heaviness of smoking [36],[37]. The significance levels of analyses of the association between individual FTND items and haplotypes are shown in Table 6 and positive scores on most items show a trend towards higher frequency of haplotype H_A_ and a lower frequency of haplotype H_C_ (Tables S5 and S6). Nominally significant results were obtained only for FTND item 4 (cigarettes per day) and FTND item 5 (smoking frequently during the first hours after waking). H_A_ vs. H_C_ associations with FTND items were tested within age of onset condition using chi-square for dichotomous items. For items 1 and 4, which have 4 ordered response options, Cochran's Test of Linear Trend was used to test the hypothesis that haplotype proportions change linearly across the response options. The only nominally significant results were obtained in the early onset condition for cigarettes per day (Item 4), Cochran's Test of Linear Trend (1, N = 1499) = 16.6, p<0.00005, and for early morning smoking (Item 5), χ2 (1, N = 1142) = 4.01, p<0.05. In sum, the pattern of findings suggests that multiple items contribute, albeit modestly in some cases, to the measurement of nicotine dependence as it is associated with haplotypes. Since the items of the FTND are not highly correlated with one another, the additive effects of the different items have the potential to yield orthogonal variance and a more comprehensive assessment of nicotine dependence than is available via only a subset of items [2].

**Table 6:** Associations between Haplotypes and Individual FTND Items.

FTND Item^a^	*p*-values^b^			
	Inter	All	Early	Late
1. How soon after you wake up do you smoke your first cigarette?	0.56	0.18	0.19	0.38
2. Do you find it difficult to refrain from smoking in places where it is forbidden?	0.84	0.63	0.68	0.90
3. Which cigarette would you hate most to give up, first one in the morning?	0.93	0.42	0.53	0.58
4. How many cigarettes per day did/do you smoke?	0.18	0.0001	0.0001	0.07
5. Do you smoke more frequently during the first hours after waking than during the rest of the day?	0.28	0.08	0.05	0.58
6. Do you smoke if you are so ill that you are in bed most of the day?	0.25	0.24	0.08	0.83

a *p*-values for items 1 and 4 are from GLM tests in which the 4 levels of the item responses were taken to be an ordinal variable. For items 2, 3, 5 and 6, *p*-values are from logistic regression analyses.

b “Inter” = the interaction between haplotype and age of onset, “All” = all subjects, “Early” = early onset smokers only, and “Late” = late onset smokers only. For GLM analyses, the *p*-value was obtained in a test across H_A_, H_B_, and H_C_. For logistic regression analyses, the *p*-value was for the H_A_ vs. H_C_ effect.

## Discussion

These results show that *CHRNA5-A3-B4* haplotypes are consistently related to severity of nicotine dependence among long-term smokers of European-American descent who began daily smoking at or before age 16 but not among those who began smoking daily after age 16. The robustness of the genotype by age of onset interaction is supported by the fact that there was a significant interaction between the two variables in logistic regression analyses and by the fact that significant associations between genetic variants and dependence were specific to early onset smokers in all three cohorts. Both human and animal research shows that early vs. late smoking or nicotine exposure is associated with more severe nicotine dependence, or greater nicotine self-administration, manifested in adulthood [25], [45]–[47] and that adolescence is a period of heightened sensitivity to nicotine reward as well as decreased sensitivity to nicotine's aversive actions [47]–[50]. Animal research suggests possible mechanisms for this effect related to persistent changes in brain structure and function. For instance, significantly greater high affinity nicotinic receptor binding is observed in the midbrain and striatum of adolescent versus adult onset nicotine-self-administering rats [51], indicating receptor up-regulation. Also, nicotine exerts greater differential mRNA expression effects on α5, α6 and β2 nAChR transcripts and on genes that influence neuroplasticity (e.g., *arc*) when it is administered to adolescent, as opposed to adult rats [25],[52].

The most noteworthy coding SNP contained in the H_A_ LD structure is rs16969968, a nonsynonymous α5 coding variant (p.Asp398Asn) resulting in substitution of a negatively charged residue within the M3–M4 intracellular loop, a region thought to be involved with receptor trafficking. The local amino acid context surrounding the p.Asp398Asn variant also displays accelerated protein evolution (higher lineage-specific Ka/Ks) in primate lineages within a class of genes related to nervous system development [53]. In mouse brain, α5 is a widely distributed minor subunit within heteromeric brain nAChRs, and incorporation of an α5 subunit into brain nAChRs leads to changes in receptor-level function [54]. The association of H_A_ and the p.Asp398Asn variant with a nicotine dependence phenotype in humans suggests further research, such as an engineered mouse model, to explore the functional role and developmental expression of these variants within the process leading to chronic nicotine dependence. Both rs16969968 and rs1051730, a synonymous α3 variant, are in nearly complete linkage disequilibrium and, along with the H_A_ LD structure, occur at significantly higher frequencies in European-American populations based on HapMap allele frequencies.

In-depth haplotype analysis also revealed a protective effect of haplotype H_C_. Multiple non-coding variants are contained within the H_C_ LD structure; therefore, potential functional variants may indirectly affect receptor function through developmental and/or cell-specific expression of α5, α3 or β4 levels. The risk versus protective effects of H_A_ and H_C_ reinforce an emerging theme in complex genetics that common and rare alleles can display a range of protective, neutral and susceptibility effects [55]. The magnitude of effects reported here (haplotype frequency shifts ∼10%, haplotype-specific odds ratios = 1.5) fall within the range of other common variant effects influencing complex diseases, such as diabetes [56], coronary artery disease [56] [57], psoriasis [58] and inflammatory bowel disease [59]

In our European-American population, we did not find evidence of significant associations (*P*<0.05 in early or late onset smokers) among the four *CHRNA4* SNPs in common with our study (rs2273504, rs2236196, rs1044396, rs1044397; Table S3) and previous candidate gene association studies of nicotine dependence in Chinese men [5], and females of European-American and African-American descent [6]. A follow-up candidate gene study [9] to a genome-wide association design [8] with a definition of no dependence (FTND = 0) in controls and a FTND>3 for dependence in cases of European descent, identified significant associations within *CHRNB3* and the *CHRNA5-A3-B4* cluster. Although that study [9] and the current study employed tagging SNPs for similar LD bins, the current study did not find any association between nicotine dependence and *CHRNB3* SNPs. However, similar LD bins within the *CHRNA5-A3-B4* cluster showed a significant risk effect in both studies, including the nonsynonymous α5 rs16969968 SNP, even with the substantial difference in FTND criteria. Using similar criteria for low and high dependence as our study, a previous study [7] also reported a suggestive association (primary *P* = 0.08) of the *CHRNA5-A3-B4* cluster in a small sample population of 242 Israeli women. A recent follow-up candidate gene analysis using genome-wide association data from three European populations data suggested a risk effect of *CHRNA5-A3-B4* locus for cigarettes per day regularly smoked [32].

Subsequent to submission of this article, a GWA study using smoking quantity (cigarettes per day, CPD) as a measure of nicotine dependence observed an association of rs1051730 with CPD at genome-wide significance levels in a large Icelandic population. That study used FTND item 4 as their measure of cigarettes per day. Table S7 shows the comparison of our study and the Icelandic study using cigarettes per day as the one, in common, phenotype and rs1051730 as the one, in common, genotype. The trend in the frequency of the rs1051730 T allele is generally similar, except for the 1–10 cigarettes per day group in the Icelandic study. Clearly, their population of smokers differs from the UT-WI-LHS smokers, which is understandable since our study preferentially enrolled long-term heavy smokers who had lung disease (UT-LHS) or sought cessation treatment (WI).

Two other GWAS reports using lung cancer case-control designs, reported that rs1051730 exceeded genome-wide significance levels for association with lung cancer but having failed to measure significant associations with smoking behavior, concluded that the effect on lung cancer risk was independent of smoking behavior. Our analysis demonstrating that strong associations between *CHRNA5-A3-B4* variants and nicotine dependence are seen only amongst smokers who began daily smoking relatively early in life, coupled with the detailed molecular definition of the *CHRNA5-A3-B4* haplotype structures generated from resequencing, supports the hypothesis that the disease outcome effects of rs1051730, and other surrogate markers for Haplotype A, are mediated by nicotine addiction. In our opinion, the biological link between nicotinic receptor variants and smoking behavior is more plausible than a direct effect of these ion channels on lung cancer susceptibility. The association of rs1051730 with lung cancer may therefore be due to disease mortality related to long-term, persistent smoking caused by severe nicotine addiction [60],[61].

In summary, we have demonstrated that major *CHRNA5-A3-B4* haplotypes identify countervailing susceptibility (H_A_) and protective (H_C_) determinants for long-term nicotine dependence. A substantial shift in haplotype frequency (A vs. C = 10%) and diplotype frequency (AA vs. BC = 17%, AA vs. CC = 27%) is observed when age of exposure to nicotine is used to define an at-risk subpopulation. Identifying this interaction of a common genetic risk factor with age of daily smoking onset among the complexity of factors that influence nicotine addiction indicates how genetics can augment public health approaches to the problem of smoking-related illness, because the risk is amenable to intervention. Identification of genetically high-risk individuals who would benefit from proactive interventions, such as adolescent education and cessation clinics, may result in a population with a lower rate of adult nicotine addiction.

## Materials and Methods

### Subjects

The UT cohort was made up of respondents to community advertising for persons who had smoked more than 100 cigarettes lifetime plus a subset of the originally recruited Utah LHS cohort who had follow-up phenotyping and biosample collection in Salt Lake City, UT; these Utah participants were excluded from the LHS cohort. Participants were not drawn from a psychiatric treatment population, and no medical or behavioral treatments were offered as part of the study. UT volunteers were not excluded simply because they had a lifetime diagnosis of psychosis or Bipolar Disorder, but they were excluded if their current mental status made it impossible for them to complete the questionnaires or interviews.

WI participants were recruited by media advertisements and had to be current smokers who were motivated to quit smoking, smoked more than 9 CPD, and produced a breath sample with carbon monoxide (CO) >9 ppm at baseline. Exclusion criteria included evidence of psychosis history based on the Prime-MD structured psychiatric interview [62], significant alcohol abuse based on the Michigan Alcoholism Screening Test [MAST] [63] and clinically significant depression symptoms based on the CES-D[64]. DNA extraction for the Wisconsin subjects was performed by the Wisconsin State Laboratory of Hygiene. Study procedures were approved by the institutional review boards at the University of Wisconsin and the University of Utah. All LHS participants had COPD compared to 62% of the UT cohort. COPD was not assessed in the WI cohort, but given the younger age of those participants, it is likely that relatively few had developed COPD yet. All LHS and WI participants were smoking at the time they participated in the study, but 43% of the UT cohort had been tobacco abstinent for the previous two years. The LHS subjects were restricted to European Americans for whom DNA was available. De-identified demographic and phenotypic data was provided from the LHS limited access dataset by the NIH/NHLBI.

The cohorts differed in age, sex, age of onset of daily smoking, percent smoking daily by age 16, cigarettes smoked/day (CPD), and dichotomized Fagerstrom Test of Nicotine Dependence (FTND) score, but they did not differ in mean FTND (Table S1). FTND scores of 0–4 were designated low dependence and scores of 6–10 were designated high dependence. Omitting mid-range FTND scores eliminated 540 participants (19%) from further analyses. The number eliminated by cohort was: LHS, 392 (20%); UT, 73 (15%); and WI, 75 (19%). Thus, the final sample used for FTND association tests comprised 1551 LHS participants, 413 UT participants, and 323 WI participants (total N = 2287). Some participants (N = 692) in the LHS cohort were missing one FTND item (item 5, a 1-point item), so the N for FTND score for this cohort is 1251. In the 692 cases missing one item, those with scores on the remaining 5 items of 0–3 were assigned to the low dependence group, those with scores of 6–9 were assigned to the high dependence group, and those with scores of 4 or 5 (N = 392) were omitted.

### Resequencing of nAChR Genes, SNP Selection, and Genotyping

DNA samples were obtained from all subjects. The resequencing sample comprised 144 smokers identified by a multiple predictor model with unit weighting as being either high or low on a broad range of dependence measures (e.g., relapse likelihood, withdrawal severity; N = 144) and an additional group of lifetime nonsmokers (N = 48). The University of California Santa Cruz genome browser was used to extract positions of proximal promoters and RefSeq exons for *CHRNA2*, *CHRNA3*, *CHRNA4*, *CHRNA5*, *CHRNA6*, *CHRNA7* (unique 5′ only), *CHRNA9*, *CHRNA10*, *CHRNB2*, *CHRNB3* and *CHRNB4*. Exons, genomic sequences conserved in mouse, and ∼1 kb of 5′ and 3′ flanking regions were targeted by DNA sequencing, as previously described [65]. The resequencing scan surveyed a total range of 56,620 nts. in each of 192 individuals of Northern European descent; survey coordinates from NCBI Build 35 for genomic ranges surveyed with a Phrap quality score of >30 are: *CHRNA2* [Chr. 8] 27374096::27375538, 27376211::27377643, 27380318::27381167, 27382561::27383658, 27384018::27384835, 27392219::27392738 nts.; *CHRNA5-CHRNA3-CHRNB4* [Chr. 15] 76644811::76645440, 76651089::76651811, 76659985::76660542, 76665576::76666420, 76667214::76668294, 76668898::76670309, 76672128::76676199, 76680573::76681761, 76696115::76696969, 76697824::76698624, 76700082::76700676, 76703348::76704858, 76708170::76709554, 76710234::76710990, 76713688::76715398, 76720101::76721040 nts.; *CHRNA4* [Chr. 20] 61446755::61447910, 61448032::61448901, 61451297::61452331, 61457596::61458129, 61457956::61458391, 61461159::61461713 nts.; *CHRNB3-CHRNA6* [Chr. 8] 42671565::42672135, 42682775::42683415, 42684268::42684976, 42704488::42705250, 42705850::42706962, 42710633::42711239, 42721158::42721408, 42726921::42727704, 42730060::42731305, 42730845::42731665, 42733016::42733750, 42739105::42739734, 42742436::42743042 nts.; *CHRNA9* [Chr. 4] 40177941::40178826, 40178370::40179265, 40179840::40180696, 40191528::40192719, 40192719::40198282 nts.; *CHRNA10* [Chr. 11] 3643245::3644701, 3644675::3645949, 3647081::3647752, 3647274::3648079, 3648702::3649607 nts.; *CHRNB2* [Chr. 1] 151353228::151353745, 151354953::151356224, 151356569::151357823, 151361153::151362332 nts.

Forward and reverse PCR primer sequences were chosen from the publicly available genomic sequence, and PCR amplification was carried out in 25 µl reaction volumes using standard techniques (primer sequences are available from the authors upon request). Amplification primers and unincorporated nucleotides were removed using ExoSAP-IT (USB, Cleveland, Ohio). For sequencing, internal primers were used, and cycle sequencing was carried out using Applied Biosystems BigDye terminator chemistry. Cycling was done with an initial denaturation at 96°C for 30 sec.; followed by 45 cycles of 96°C for 10 sec., 50°C for 5 sec., 60°C for 4 min. Upon completion of sequencing, 20 µl of 62.5% EtOH/1M KOAc, pH 4.5 was added to each reaction and the sequence plates were centrifuged at 4000 rpm at 4°C for 45 min. The samples were resuspended in 15 ul of formamide and electrophoresed on an ABI 3700 capillary instrument. Sequence trace files were evaluated using the *Phred*, *Phrap* and *Consed* programs, and potential variants were identified by using the *PolyPhred* program [66],[67]. Single nucleotide polymorphisms (SNPs) were verified by manual evaluation of the individual forward and reverse sequence traces. In addition, all sequence assemblies were manually scanned for insertions, deletions and polymorphic positions undetected by *PolyPhred*. Tag SNPs were selected using the ldSelect algorithm [68] with an r^2^ cutoff value of 0.64 on all SNPs with a minor allele frequency >5%.

Genotyping was carried out using one of two different methods: an oligonucleotide ligation, PCR assay for simultaneous genotyping of 48 single nucleotide polymorphisms (SNPs) in a multiplexed, 384-well plate method, SNPlex (Applied Biosystems), and a single-step homogeneous SNP genotyping using a 5′-nuclease assay, TaqMan (Applied Biosystems). The SNPlex assay, pooling 48 different SNPs into a single genotyping assay, was applied to DNA samples processed in 384-well plate format through an automated protocol which included: SNP-specific oligonucleotide ligation, PCR amplification, immobilization, hybridization, elution, and separation and fluorescent detection on an ABI 3730xl capillary instrument. For all SNPs genotyped by SNPlex, the call rate on the 932 individuals in the UT and WI cohort was 99.26% after 35 individuals were excluded for low genotyping rates (>10% missing genotypes). TaqMan genotyping was processed in 384-well plate format on the five SNPs in the combined UT-WI-LHS cohorts, and the call rate on 2,006 individuals was 99.42% after 29 individuals were excluded for missing genotypes. For the five SNPs genotyped by TaqMan and SNPlex methods in 236 individuals, there were three discordant calls, all in a single individual. For the 87 SNPs genotyped by SNPlex and resequencing in the subset of 192 individuals, the concordance rate was 99.7%.

### Statistical Analysis

SNP genotypes were evaluated for standard summary measures including genotyping rates, allele and genotype frequencies, and Hardy-Weinberg equilibrium tests. Standard case/control allelic *χ* ^2^ analyses were used to test the association of SNPs with dichotomized FTND, as well as the Cochran-Armitage trend test using the computer program, PLINK [69]. Empirical significance levels of allelic tests were evaluated by phenotype-genotype permutation testing using the PLINK adaptive mode. The logistic regression analyses reported in Tables 3–4 and Figure 2 were computed using SYSTAT 10.2 (Richmond, CA: SYSTAT Software Inc.). Dummy coding was used for haplotype and sex, while ordinal coding was used for age of onset of daily smoking. Haplotype estimation and individual assignment were carried out on genotypic data using fastPHASE [70], and independently evaluated using the EM algorithm implemented in SNPHAP (http://www-gene.cimr.cam.ac.uk/clayton/software). Haplotype-based association analyses for omnibus and haplotype-specific tests were carried out using PLINK. To evaluate potential population stratification effects, the UT and WI cohorts were analyzed for population admixture using STRUCTURE [71] on 94 non-related loci and an assumed population of 2. No significant admixture was observed, supporting the European American self report. Also, cohorts did not differ in *CHRNA5-A3-B4* haplotype frequency (χ2 _(6, N = 5654)_ = 8.2, *P* = 0.22, see Table 2), suggesting they were drawn from the same population.

## Supporting Information

Table S1 Demographics and smoking history by cohort.
(0.01 MB PDF)

Table S2 Single nucleotide polymorphism discovery by genomic resequencing in α-like nAChR (α2, α3, α4, α5, α6, α9, and α10) and β-like nAChR (β2, β3, and β4) subunit genes.
(0.05 MB PDF)

Table S3 Association of nicotinic receptor variants using dichotomized FTND Scores within age of onset of daily smoking in the combined UT and WI cohorts.
(0.23 MB DOC)

Table S4 Results of logistic regression analyses in which Haplotype C was the reference condition and two alternative criteria for low and high nicotine dependence were used (0–4 vs. 5–10 and 0–5 vs. 6–10).
(0.01 MB PDF)

Table S5 Frequency of individual *CHRNA5-A3* SNPs in the UT-WI-LHS cohorts according to individual FTND items.
(0.12 MB DOC)

Table S6 Frequency of Haplotypes A, B and C in the UT-WI-LHS cohorts according to individual FTND items.
(0.01 MB PDF)

Table S7 rs1051730 genotype and FTND item 4 comparison between combined early and late onset UT-WI-LHS smokers versus Icelandic smokers.
(0.01 MB PDF)

## References

[1] Breslau N, Johnson EO (2000). Predicting smoking cessation and major depression in nicotine-dependent smokers.. Am J Public Health.

[2] Piper ME, McCarthy DE, Baker TB (2006). Assessing tobacco dependence: a guide to measure evaluation and selection.. Nicotine Tob Res.

[3] U.S. Department of Health and Human Services (2004). The health consequences of smoking: A report of the Surgeon General.

[4] Li MD, Cheng R, Ma JZ, Swan GE (2003). A meta-analysis of estimated genetic and environmental effects on smoking behavior in male and female adult twins.. Addiction.

[5] Feng Y, Niu T, Xing H, Xu X, Chen C (2004). A common haplotype of the nicotine acetylcholine receptor alpha 4 subunit gene is associated with vulnerability to nicotine addiction in men.. Am J Hum Genet.

[6] Li MD, Beuten J, Ma JZ, Payne TJ, Lou XY (2005). Ethnic- and gender-specific association of the nicotinic acetylcholine receptor alpha4 subunit gene (CHRNA4) with nicotine dependence.. Hum Mol Genet.

[7] Greenbaum L, Kanyas K, Karni O, Merbl Y, Olender T (2006). Why do young women smoke? I. Direct and interactive effects of environment, psychological characteristics and nicotinic cholinergic receptor genes.. Mol Psychiatry.

[8] Bierut LJ, Madden PA, Breslau N, Johnson EO, Hatsukami D (2007). Novel genes identified in a high-density genome wide association study for nicotine dependence.. Hum Mol Genet.

[9] Saccone SF, Hinrichs AL, Saccone NL, Chase GA, Konvicka K (2007). Cholinergic nicotinic receptor genes implicated in a nicotine dependence association study targeting 348 candidate genes with 3713 SNPs.. Hum Mol Genet.

[10] Breslau N, Peterson EL (1996). Smoking cessation in young adults: age at initiation of cigarette smoking and other suspected influences.. Am J Public Health.

[11] Chassin L, Presson CC, Rose JS, Sherman SJ (1996). The natural history of cigarette smoking from adolescence to adulthood: demographic predictors of continuity and change.. Health Psychol.

[12] Chen J, Millar WJ (1998). Age of smoking initiation: implications for quitting.. Health Rep.

[13] Lando HA, Thai DT, Murray DM, Robinson LA, Jeffery RW (1999). Age of initiation, smoking patterns, and risk in a population of working adults.. Prev Med.

[14] Broms U, Silventoinen K, Lahelma E, Koskenvuo M, Kaprio J (2004). Smoking cessation by socioeconomic status and marital status: the contribution of smoking behavior and family background.. Nicotine Tob Res.

[15] Kandel DB, Hu MC, Griesler PC, Schaffran C (2007). On the development of nicotine dependence in adolescence.. Drug Alcohol Depend.

[16] Chen X, Stanton B, Shankaran S, Li X (2006). Age of smoking onset as a predictor of smoking cessation during pregnancy.. Am J Health Behav.

[17] Hymowitz N, Cummings KM, Hyland A, Lynn WR, Pechacek TF (1997). Predictors of smoking cessation in a cohort of adult smokers followed for five years.. Tobacco Control.

[18] John U, Meyer C, Hapke U, Rumpf HJ (2004). Nicotine dependence and lifetime amount of smoking in a population sample.. Eur J Public Health.

[19] Grant BF (1998). Age at smoking onset and its association with alcohol consumption and DSM-IV alcohol abuse and dependence: results from the National Longitudinal Alcohol Epidemiologic Survey.. J Subst Abuse.

[20] John U, Meyer C, Rumpf HJ, Schumann A, Hapke U (2005). Consistency or change in nicotine dependence according to the Fagerstrom Test for Nicotine Dependence over three years in a population sample.. J Addict Dis.

[21] Pergadia ML, Heath AC, Martin NG, Madden PA (2006). Genetic analyses of DSM-IV nicotine withdrawal in adult twins.. Psychol Med.

[22] Xian H, Scherrer JF, Eisen SA, Lyons MJ, Tsuang M (2007). Nicotine dependence subtypes: Association with smoking history, diagnostic criteria and psychiatric disorders in 5440 regular smokers from the Vietnam Era Twin Registry.. Addict Behav.

[23] Trauth JA, Seidler FJ, Ali SF, Slotkin TA (2001). Adolescent nicotine exposure produces immediate and long-term changes in CNS noradrenergic and dopaminergic function.. Brain Res.

[24] Slotkin TA (2002). Nicotine and the adolescent brain: Insights from an animal model.. Neurotoxicology and Teratology.

[25] Adriani W, Spijker S, Deroche-Gamonet V, Laviola G, Le Moal M (2003). Evidence for enhanced neurobehavioral vulnerability to nicotine during periadolescence in rats.. J Neurosci.

[26] Cruz FC, Delucia R, Planeta CS (2005). Differential behavioral and neuroendocrine effects of repeated nicotine in adolescent and adult rats.. Pharmacol Biochem Behav.

[27] Schochet TL, Kelley AE, Landry CF (2004). Differential behavioral effects of nicotine exposure in adolescent and adult rats.. Psychopharmacology (Berl).

[28] Dani JA, Heinemann S (1996). Molecular and cellular aspects of nicotine abuse.. Neuron.

[29] Besson M, Granon S, Mameli-Engvall M, Cloez-Tayarani I, Maubourguet N (2007). Long-term effects of chronic nicotine exposure on brain nicotinic receptors.. Proc Natl Acad Sci U S A.

[30] Picciotto MR, Zoli M, Rimondini R, Lena C, Marubio LM (1998). Acetylcholine receptors containing the beta2 subunit are involved in the reinforcing properties of nicotine.. Nature.

[31] Tapper AR, McKinney SL, Nashmi R, Schwarz J, Deshpande P (2004). Nicotine activation of alpha4* receptors: sufficient for reward, tolerance, and sensitization.. Science.

[32] Berrettini W, Yuan X, Tozzi F, Song K, Francks C (2008). Alpha-5/alpha-3 nicotinic receptor subunit alleles increase risk for heavy smoking.. Mol Psychiatry.

[33] Amos CI, Wu X, Broderick P, Gorlov IP, Gu J (2008). Genome-wide association scan of tag SNPs identifies a susceptibility locus for lung cancer at 15q25.1.. Nat Genet.

[34] Hung RJ, McKay JD, Gaborieau V, Boffetta P, Hashibe M (2008). A susceptibility locus for lung cancer maps to nicotinic acetylcholine receptor subunit genes on 15q25.. Nature.

[35] Thorgeirsson TE, Geller F, Sulem P, Rafnar T, Wiste A (2008). A variant associated with nicotine dependence, lung cancer and peripheral arterial disease.. Nature.

[36] Heatherton TF, Kozlowski LT, Frecker RC, Fagerstrom KO (1991). The Fagerstrom Test for Nicotine Dependence: a revision of the Fagerstrom Tolerance Questionnaire.. Br J Addict.

[37] Pomerleau CS, Carton SM, Lutzke ML, Flessland KA, Pomerleau OF (1994). Reliability of the Fagerstrom Tolerance Questionnaire and the Fagerstrom Test for Nicotine Dependence.. Addict Behav.

[38] Anthonisen NR, Connett JE, Kiley JP, Altose MD, Bailey WC (1994). Effects of smoking intervention and the use of an inhaled anticholinergic bronchodilator on the rate of decline of FEV1. The Lung Health Study.. Jama.

[39] Hudmon KS, Pomerleau CS, Brigham J, Javitz H, Swan GE (2005). Validity of retrospective assessments of nicotine dependence: a preliminary report.. Addict Behav.

[40] Pergadia ML, Heath AC, Agrawal A, Bucholz KK, Martin NG (2006). The implications of simultaneous smoking initiation for inferences about the genetics of smoking behavior from twin data.. Behav Genet.

[41] Huerta M, Chodick G, Balicer RD, Davidovitch N, Grotto I (2005). Reliability of self-reported smoking history and age at initial tobacco use.. Prev Med.

[42] Chabrol H, Niezborala M, Chastan E, de Leon J (2005). Comparison of the Heavy Smoking Index and of the Fagerstrom Test for Nicotine Dependence in a sample of 749 cigarette smokers.. Addict Behav.

[43] Fagerstrom KO, Kunze M, Schoberberger R, Breslau N, Hughes JR (1996). Nicotine dependence versus smoking prevalence: comparisons among countries and categories of smokers.. Tob Control.

[44] Ferguson JA, Patten CA, Schroeder DR, Offord KP, Eberman KM (2003). Predictors of 6-month tobacco abstinence among 1224 cigarette smokers treated for nicotine dependence.. Addictive Behaviors.

[45] Belluzzi JD, Wang R, Leslie FM (2005). Acetaldehyde enhances acquisition of nicotine self-administration in adolescent rats.. Neuropsychopharmacology.

[46] Levin ED, Rezvani AH, Montoya D, Rose JE, Swartzwelder HS (2003). Adolescent-onset nicotine self-administration modeled in female rats.. Psychopharmacology (Berl).

[47] Vastola BJ, Douglas LA, Varlinskaya EI, Spear LP (2002). Nicotine-induced conditioned place preference in adolescent and adult rats.. Physiol Behav.

[48] Adriani W, Macri S, Pacifici R, Laviola G (2002). Peculiar vulnerability to nicotine oral self-administration in mice during early adolescence.. Neuropsychopharmacology.

[49] Belluzzi JD, Lee AG, Oliff HS, Leslie FM (2004). Age-dependent effects of nicotine on locomotor activity and conditioned place preference in rats.. Psychopharmacology (Berl).

[50] Shram MJ, Funk D, Li Z, Le AD (2006). Periadolescent and adult rats respond differently in tests measuring the rewarding and aversive effects of nicotine.. Psychopharmacology (Berl).

[51] Levin ED, Lawrence SS, Petro A, Horton K, Rezvani AH (2007). Adolescent vs. adult-onset nicotine self-administration in male rats: duration of effect and differential nicotinic receptor correlates.. Neurotoxicol Teratol.

[52] Schochet TL, Kelley AE, Landry CF (2005). Differential expression of arc mRNA and other plasticity-related genes induced by nicotine in adolescent rat forebrain.. Neuroscience.

[53] Dorus S, Vallender EJ, Evans PD, Anderson JR, Gilbert SL (2004). Accelerated evolution of nervous system genes in the origin of Homo sapiens.. Cell.

[54] Brown RW, Collins AC, Lindstrom JM, Whiteaker P (2007). Nicotinic alpha5 subunit deletion locally reduces high-affinity agonist activation without altering nicotinic receptor numbers.. J Neurochem.

[55] Li M, Atmaca-Sonmez P, Othman M, Branham KE, Khanna R (2006). CFH haplotypes without the Y402H coding variant show strong association with susceptibility to age-related macular degeneration.. Nat Genet.

[56] (2007). Genome-wide association study of 14,000 cases of seven common diseases and 3,000 shared controls.. Nature.

[57] Samani NJ, Erdmann J, Hall AS, Hengstenberg C, Mangino M (2007). Genomewide association analysis of coronary artery disease.. N Engl J Med.

[58] Cargill M, Schrodi SJ, Chang M, Garcia VE, Brandon R (2007). A large-scale genetic association study confirms IL12B and leads to the identification of IL23R as psoriasis-risk genes.. Am J Hum Genet.

[59] Duerr RH, Taylor KD, Brant SR, Rioux JD, Silverberg MS (2006). A genome-wide association study identifies IL23R as an inflammatory bowel disease gene.. Science.

[60] Flanders WD, Lally CA, Zhu BP, Henley SJ, Thun MJ (2003). Lung cancer mortality in relation to age, duration of smoking, and daily cigarette consumption: results from Cancer Prevention Study II.. Cancer Res.

[61] Knoke JD, Shanks TG, Vaughn JW, Thun MJ, Burns DM (2004). Lung cancer mortality is related to age in addition to duration and intensity of cigarette smoking: an analysis of CPS-I data.. Cancer Epidemiol Biomarkers Prev.

[62] Spitzer RL, Williams JB (1994). American psychiatry's transformation following the publication of DSM-III.. Am J Psychiatry.

[63] Selzer ML, Vinokur A, van Rooijen L (1975). A self-administered Short Michigan Alcoholism Screening Test (SMAST).. J Stud Alcohol.

[64] Radloff LS (1977). The CES-D Scale: A self-report depression scale for research in the general population.. Journal of Applied Psychological Measures.

[65] Flanigan KM, von Niederhausern A, Dunn DM, Alder J, Mendell JR (2003). Rapid direct sequence analysis of the dystrophin gene.. Am J Hum Genet.

[66] Livingston RJ, von Niederhausern A, Jegga AG, Crawford DC, Carlson CS (2004). Pattern of sequence variation across 213 environmental response genes.. Genome Res.

[67] Stephens M, Sloan JS, Robertson PD, Scheet P, Nickerson DA (2006). Automating sequence-based detection and genotyping of SNPs from diploid samples.. Nat Genet.

[68] Carlson CS, Eberle MA, Rieder MJ, Yi Q, Kruglyak L (2004). Selecting a maximally informative set of single-nucleotide polymorphisms for association analyses using linkage disequilibrium.. Am J Hum Genet.

[69] Purcell S, Neale B, Todd-Brown K, Thomas L, Ferreira MA (2007). PLINK: a tool set for whole-genome association and population-based linkage analyses.. Am J Hum Genet.

[70] Scheet P, Stephens M (2006). A fast and flexible statistical model for large-scale population genotype data: applications to inferring missing genotypes and haplotypic phase.. Am J Hum Genet.

[71] Pritchard JK, Stephens M, Donnelly P (2000). Inference of population structure using multilocus genotype data.. Genetics.

