# Supplementary material for: A Candidate Gene Approach Identifies the CHRNA5-A3-B4 Region as a Risk Factor for Age-Dependent Nicotine Addiction
Source: PLoS Genet. 2008 Jul 11;4(7):e1000125. doi: 10.1371/journal.pgen.1000125 (PMC2442220; doi:10.1371/journal.pgen.1000125)
Supplement: Table S1 — Demographics and smoking history by cohort. (0.01 MB PDF) [file pgen.1000125.s001.pdf]

Table S1. Demographics and smoking history by cohort.

**Variable**	**Cohort**			***p*-value**
	LHS<br>(N = 1943)	UT<br>(N = 486)	WI<br>(N = 398)	
Age (years)	48.5 (6.8)	59.3 (10.5)	42.7 (10.6)	<0.001
Sex (% female)	38%	42%	55%	<0.001
Onset of daily smoking (years)	17.4 (3.9)	18.0 (4.9)	16.2 (3.5)	<0.001
Daily smoking by age 16 (%)	44%	39%	62%	<0.001
Cigarettes per day	29.5 (13.9)	27.7 (15.6)	23.0 (9.2)	<0.001
FTND score	5.8 (2.2)	5.6 (2.3)	5.6 (2.1)	<0.001
FTND46 (% low)	32%	37%	37%	0.28

For quantitative variables, means and SDs (in parentheses) are shown with, and *p*-values are from ANOVAs. For categorical variables, percentages are shown and *p*-values are from chi-square tests. The FTND46 dichotomy was derived form FTND scores of 0-4 assigned to the low dependence condition and scores of 6-10 assigned to the high dependence condition with scores = 5 omitted.
