# Supplementary material for: A Candidate Gene Approach Identifies the CHRNA5-A3-B4 Region as a Risk Factor for Age-Dependent Nicotine Addiction
Source: PLoS Genet. 2008 Jul 11;4(7):e1000125. doi: 10.1371/journal.pgen.1000125 (PMC2442220; doi:10.1371/journal.pgen.1000125)
Supplement: Table S2 — Single nucleotide polymorphism discovery by genomic resequencing in α-like nAChR (α2, α3, α4, α5, α6, α9, and α10) and β-like nAChR (β2, β3, and β4) subunit genes. (0.05 MB PDF) [file pgen.1000125.s002.pdf]

Table S2. Single Nucleotide Polymorphism discovery by genomic resequencing in seven α-like nAChR subunits (α2, α3, α4, α5, α6, α9, and α10) and three β-like nAChR subunits (β2, β3, and β4). Resequencing was performed on 192 individuals of Northern European descent. The individuals were chosen based on a high (n=72) and low (n=72) dependence scale and nonsmokers (n=48). Chromosomal positions ('Position') are based on the NCBI Human Genome Build 35 (May 2004). 'Imp.' designates annotation of SNP location, and 'Major' – 'Minor' allele assignments are based on the forward strand of the chromosome. Linkage disequilibrium groups ('LD Group') were determined using the ldSelect algorithm [68] at a threshold r^2 value of 0.64 on the 192 individuals across a gene or gene group (based on close proximity) and assigned a unique number. Genes are ordered by ascending chromosome number. The *P*-values are Fisher exact allele tests of high vs. low nicotine dependence with minor allele frequencies shown for these groups. The 'Genotyped' column indicates polymorphisms typed on the larger cohort.

**Gene: Chromosome**	**Position**	**Imp.**	**Major**	**Minor**	**LD Group**	**Freq. High**	**Freq. Low**	**P value**	**refSNP ID**	**Genotyped**
**CHRNB2: Chr 1**	151353472	UTR	C	A		0.01	0.01	-	rs56205574	
	151353541	UTR	C	T	2	0.11	0.10	0.84	rs2280781	+
	151354912	intronic	A	T		0.04	0.02	0.45	rs8192484	
	151355288	intronic	G	A		0.01	0.01	-	rs55698310	
	151357563	non	G	C		0.01	0.01	-	rs55685423	
	151357605	synon	G	A		0.01	0.01	-	rs55857552	
	151357724	intronic	G	T	2	0.12	0.07	0.21	rs4845378	
	151357735	intronic	G	A		0.01	0.01	-	rs56146528	
	151361594	UTR	C	G		0.07	0.09	0.67	rs2072659	+
	151361794	UTR	C	T	1	0.30	0.20	0.07	rs2072660	+
	151361953	UTR	G	A	2	0.30	0.22	0.17	rs2072661	
	151362019	UTR	C	T	3	0.06	0.08	0.49	rs4292956	+

Gene: Chromosome	Position	Imp.	Major	Minor	LD Group	Freq. High	Freq. Low	P value	refSNP ID	Genotyped
	151362065	UTR	C	T	3	0.06	0.06	1.00	rs45490696	
**CHRNA9: Chr 4**										
	40178152	flank	C	A	4	0.30	0.28	0.89	rs372211	
	40178176	flank	A	T	4	0.30	0.28	0.79	rs4469116	+
	40178236	flank	A	T	4	0.30	0.28	0.70	rs4359945	
	40178241	flank	G	A		0.00	0.01	-	rs55725809	
	40178244	flank	C	T		0.01	0.03	0.68	rs6544304	
	40178451	synon	C	T	4	0.36	0.31	0.45	rs56241474	
	40178494	intronic	C	T		0.21	0.25	0.48	rs10015231	
	40178836	synon	C	T	6	0.40	0.41	0.90	rs10022491	+
	40178872	synon	C	T	4	0.31	0.28	0.70	rs56159866	
	40178896	synon	G	A		0.01	0.03	0.45	rs55974552	
	40178905	synon	G	A		<0.01	0.01	-	rs56171884	
	40178934	intronic	G	A		<0.01	0.01	-	rs56299220	
	40179007	intronic	G	A		0.03	0.05	0.54	rs55893318	
	40179152	intronic	T	C		<0.01	<0.01	1.00	rs55947729	
	40179971	intronic	-	A		0.06	0.04	0.57	rs10419703	
	40180010	intronic	A	G		<0.01	<0.01	-	rs55698633	
	40180065	intronic	G	C		0.06	0.04	0.79	rs6447332	
	40180178	synon	T	C		0.01	0.01	-	rs55998310	
	40180502	intronic	G	A	6	0.45	0.48	0.72	rs6819385	+
	40191579	intronic	G	A	4	0.36	0.33	0.69	rs7669882	+
	40191591	intronic	C	T	4	0.36	0.33	0.69	rs6447450	
	40191691	intronic	T	C	6	0.43	0.51	0.16	rs10029872	
	40192136	synon	G	A		<0.01	0.01	-	rs55665751	
	40192176	non	T	C		0.01	<0.01	-	rs56205779	
	40192387	intronic	C	A		0.01	<0.01	-	rs55660233	
	40192468	intronic	T	A		0.03	0.02	1.00	rs56078044	
	40196666	intronic	T	-	5	0.16	0.13	0.51	rs55692533	
	40196731	intronic	C	A	5	0.16	0.13	0.51	rs56012618	
	40196743	intronic	A	G	5	0.19	0.19	1.00	rs4861323	
	40196959	non	G	A		0.01	<0.01	-	rs56210055	
	40196969	non	C	T	5	0.17	0.14	0.52	rs55633891	
	40197161	non	A	G		<0.01	<0.01	-	rs56098158	
	40197220	non	C	T		0.01	<0.01	-	rs55962300	
	40197350	non	G	A	5	0.20	0.19	0.88	rs10009228	
	40197642	UTR	T	A		0.03	0.06	0.38	rs4861327	
	40197708	UTR	G	A	4	0.31	0.27	0.52	rs6819816	

Gene: Chromosome	Position	Imp.	Major	Minor	LD Group	Freq. High	Freq. Low	P value	refSNP ID	Genotyped
	40197827	UTR	C	T		0.05	0.04	0.78	rs56291234	
**CHRNA2: Chr 8**										
	27374308	UTR	C	T	10	0.44	0.45	0.88	rs2292974	
	27374342	UTR	C	A	10	0.41	0.45	0.56	rs2292975	+
	27374461	UTR	G	A	7	0.15	0.20	0.34	rs2292976	
	27374552	UTR	G	C	7	0.14	0.20	0.34	rs2292977	
	27375209	intronic	A	G		0.27	0.29	0.87	rs735421	+
	27376443	non	G	T		<0.01	0.01	-	rs56344740	
	27376553	synon	G	A		0.03	0.01	0.45	rs56298562	
	27376814	non	G	C		<0.01	0.01	-	rs56339365	
	27376892	non	C	T		<0.01	<0.01	-	rs56371471	
	27377043	synon	G	A		<0.01	0.01	-	rs56189058	
	27377106	synon	G	A		0.02	0.01	0.62	rs56229264	
	27380739	non	T	C	9	0.48	0.48	1.00	rs891398	+
	27380761	synon	G	A	7	0.13	0.13	1.00	rs2565061	+
	27380841	intronic	C	T		0.00	0.01	1.00	rs55698463	
	27381108	intronic	G	T	7	0.14	0.15	0.87	rs2099166	
	27382758	intronic	GG	-	9	0.47	0.46	1.00	rs3832545	
	27384184	intronic	C	T	7	0.11	0.12	1.00	rs2565066	
	27384271	intronic	C	G		0.33	0.28	0.37	rs2472554	+
	27384326	intronic	G	A		0.01	0.01	-	rs55965307	
	27384428	non	G	A	7	0.13	0.13	1.00	rs2472553	+
	27384537	UTR	G	T	8	0.01	0.00	-	rs12114756	
	27384705	intronic	C	A		<0.01	0.01	-	rs55954528	
	27392419	UTR	G	T	8	0.01	<0.01	-	rs55934039	
	27392545	UTR	G	A		<0.01	0.02	0.11	rs55726427	
	27392664	UTR	C	T		<0.01	0.01	-	rs56032736	
	27392684	flank	A	T	8	0.01	<0.01	-	rs2565060	
	27392699	flank	T	C	8	0.01	<0.01	-	rs34206731	
	27392703	flank	C	T	8	0.01	<0.01	-	rs56278204	
**CHRNB3: Chr 8**										
	42671628	flank	G	T		<0.01	0.01	-	rs56279199	
	42671687	flank	G	C		0.01	0.01	-	rs41272375	
	42671790	UTR	A	G		0.19	0.25	0.31	rs4950	+
	42672020	intronic	A	T		0.01	0.02	0.36	rs55920715	
	42683143	non	T	G		<0.01	0.01	-	rs56357313	
	42684671	intronic	T	-		<0.01	0.01	-	rs55786845	
	42704851	intronic	T	C	12	0.08	0.05	0.47	rs55817013	

Gene: Chromosome	Position	Imp.	Major	Minor	LD Group	Freq. High	Freq. Low	P value	refSNP ID	Genotyped
	42706222	synon	C	T	12	0.06	0.04	0.57	rs4952	
	42706709	non	G	A		0.01	0.01	-	rs56198260	
	42706816	synon	G	C	12	0.06	0.04	0.57	rs4953	+
	42710763	intronic	G	A		<0.01	<0.01	-	rs55924223	
	42710995	UTR	G	A		0.01	0.01	-	rs56313404	
**CHRNA6: Chr 8**										
	42726938	flank	T	A	11	0.17	0.22	0.29	rs7832639	
	42726994	UTR	G	A		0.01	<0.01	-	rs55776131	
	42727068	UTR	T	C		0.03	0.00	0.06	rs41265262	
	42727243	UTR	C	T		0.01	0.00	-	rs56044754	
	42727356	UTR	G	C	11	0.21	0.28	0.17	rs2304297	
	42727538	non	C	T		0.00	0.00	-	rs55662044	
	42727720	intronic	G	A	11	0.14	0.23	0.07	rs7845663	
	42730880	stop	C	A		<0.01	0.01	-	rs56315032	
	42731021	frameshift	T	-		0.01	<0.01	-	rs56229628	
	42731443	intronic	G	A	11	0.14	0.24	0.04	rs7000412	
	42731535	intronic	C	A	11	0.14	0.24	0.05	rs7001753	
	42733291	intronic	T	C		0.00	0.01	-	rs55984829	
	42733535	intronic	C	A	11	0.16	0.25	0.08	rs892413	+
	42739158	intronic	C	G	11	0.15	0.24	0.05	rs1072003	+
	42739555	intronic	C	T		0.01	<0.01	-	rs56238139	
	42739561	intronic	C	A		0.01	0.03	0.21	rs11985288	
	42742951	flank	ATG	-		0.02	0.01	0.62	rs56376656	
**CHRNA10: Chr 11**										
	3643303	flank	G	A		0.13	0.11	0.70	rs56084560	
	3643385	flank	C	T	14	0.05	0.05	1.00	rs12269960	
	3643435	UTR	C	G		<0.01	0.01	-	rs12711988	
	3643520	UTR	C	G	16	<0.01	0.02	0.25	rs12221525	
	3643625	UTR	T	A	14	0.06	0.06	1.00	rs55798582	
	3644005	non	G	A		<0.01	0.01	-	rs2231548	
	3644050	non	A	G		<0.01	<0.01	-	rs55650071	
	3644052	non	G	T		0.01	<0.01	-	rs56054950	
	3644202	non	T	G	14	0.06	0.06	1.00	rs2231547	
	3644227	non	C	T	14	0.06	0.06	1.00	rs2231546	
	3644561	intronic	A	G	13	0.29	0.27	0.79	rs2741862	+
	3644640	intronic	TTTA	-		0.01	<0.01	0.50	rs55839829	
	3644804	intronic	G	A	13	0.36	0.35	0.88	rs2741861	
	3644856	intronic	G	C	17	<0.01	0.01	-	rs56101595	

Gene: Chromosome	Position	Imp.	Major	Minor	LD Group	Freq. High	Freq. Low	P value	refSNP ID	Genotyped
	3646686	intronic	A	G		<0.01	0.01	-	rs55836061	
	3646697	intronic	G	A		0.01	<0.01	-	rs55933520	
	3646759	intronic	G	A	13	0.01	<0.01	-	rs2741868	+
	3646854	intronic	T	C	13	0.35	0.36	0.80	rs2672211	
	3647134	non	G	T	15	0.04	0.02	0.50	rs55719530	
	3647174	intronic	T	A	15	0.04	0.02	0.50	rs56167171	
	3647338	intronic	CT	-		0.18	0.15	0.63	rs3996926	
	3647347	intronic	A	-	17	<0.01	0.01	1.00	rs55794740	
	3647439	intronic	A	C		0.01	0.04	0.45	rs11028916	
	3647450	intronic	14nts[1]	-		<0.01	<0.01	-	rs56081677	
	3647789	intronic	T	A		<0.01	0.01	-	rs55716405	
	3647913	intronic	G	C		<0.01	<0.01	-	rs55966314	
	3647993	intronic	C	T		0.18	0.12	0.23	rs2672213	+
	3648013	intronic	T	G	18	<0.01	<0.01	-	rs16942496	
	3648088	intronic	T	C	13	0.33	0.34	0.90	rs2672214	
	3649205	flank	A	G	18	<0.01	0.01	-	rs55982775	
	3649219	flank	G	T	16	<0.01	0.03	0.12	rs2231536	
	3649354	flank	C	G		<0.01	0.01	-	rs55886645	
**CHRNA5: Chr 15**										
	76644934	flank	G	A		<0.01	0.01	-	rs56182392	
	76644951	flank	T	A	21	0.22	0.22	1.00	rs503464	
	76644994	UTR	T	G	20	0.49	0.38	0.10	rs55853698	
	76645041	UTR	C	G	20	0.49	0.38	0.11	rs55781567	
	76651079	intronic	C	T		0.02	0.02	1.00	rs56219480	
	76651199	intronic	G	A		<0.01	<0.01	-	rs55758640	
	76660174	intronic	C	T	21	0.18	0.22	0.45	rs569207	+
	76660327	non	T	G		<0.01	<0.01	-	rs56351164	
	76665596	intronic	G	A	20	0.53	0.32	0.002	rs951266	
	76665620	intronic	C	T		<0.01	0.02	0.20	rs56160480	
	76665737	intronic	A	G		0.01	<0.01	-	rs56211565	
	76666023	intronic	A	C		<0.01	0.01	-	rs56257539	
	76666113	intronic	C	T		0.01	0.01	-	rs55982512	
	76666297	intronic	A	G	19	0.31	0.45	0.03	rs555018	+
	76667536	intronic	C	T	19	0.31	0.35	0.60	rs647041	
	76667632	intronic	G	C	22	0.05	0.06	0.60	rs12898919	
	76667748	non	A	G	24	0.01	0.00	-	rs56023835	
	76667807	non	G	A		0.04	0.02	0.45	rs2229961	+

[1] Major Allele: TCAGTACCCAACAG

Gene: Chromosome	Position	Imp.	Major	Minor	LD Group	Freq. High	Freq. Low	P value	refSNP ID	Genotyped
	76668142	intronic	G	A	22	0.06	0.07	0.79	rs12903575	+
	76669007	intronic	T	C		<0.01	<0.01	-	rs56186290	
	76669059	intronic	C	T		0.01	<0.01	-	rs56201623	
	76669159	intronic	T	C		0.01	0.01	-	rs56134824	
	76669276	non	C	T		0.00	0.01	-	rs55863434	
	76669980	non	G	A	20	0.50	0.34	0.008	rs16969968	+
	76673043	UTR	C	T	19	0.28	0.38	0.09	rs615470	
	76673204	UTR	G	A		<0.01	<0.01	-	rs8192483	
	76673213	UTR	G	A		<0.01	0.02	0.22	rs55783657	
	76673253	UTR	C	T	20	0.51	0.32	0.003	rs8192482	
	76673282	UTR	A	G	21	0.20	0.27	0.24	rs564585	
	76673903	flank	G	A		<0.01	0.01	-	rs55989223	
	76673947	flank	C	T		<0.01	0.01	-	rs56255741	
	76674002	flank	G	A	20	0.49	0.34	0.01	rs4887067	
**CHRNA3: Chr 15**										
	76674415	flank	A	C		0.01	<0.01	-	rs55919839	
	76674493	flank	T	G	22	0.04	0.06	0.44	rs12899226	
	76674550	flank	G	A		<0.01	0.01	-	rs55736590	
	76674770	UTR	TCTC	-	19	0.29	0.38	0.13	rs56297775	
	76674887	UTR	G	A	19	0.29	0.38	0.13	rs660652	
	76675049	UTR	G	A	19	0.29	0.38	0.13	rs472054	
	76675098	UTR	13nts[2]	-		<0.01	<0.01	-	rs55948826	
	76675213	UTR	G	A		<0.01	0.01	-	rs56148181	
	76675293	UTR	3(C)	7(C)	21	0.1	0.2	0.60	rs10637216	
	76675406	UTR	G	A		0.01	0.01	1.00	rs56113144	
	76675455	UTR	G	A		0.21	0.26	0.40	rs578776	+
	76675568	UTR	10nts[3]	-		<0.01	0.01	-	rs55820134	
	76675663	UTR	A	G	24	0.01	<0.01	-	rs56256191	
	76680842	synon	G	A		0.01	0.01	-	rs56403513	
	76680914	synon	G	A		0.01	<0.01	-	rs56179957	
	76681338	non	G	A		0.01	<0.01	-	rs56371320	
	76681394	synon	G	A	20	0.49	0.36	0.02	rs1051730	+
	76681412	synon	G	T		0.01	0.01	-	rs55958820	
	76681475	synon	A	G		0.01	0.01	-	rs8192480	
	76696453	synon	C	T		0.06	0.01	0.10	rs8192479	
	76696507	synon	C	T	19	0.28	0.37	0.13	rs3743075	

[2] Major Allele: ATGCTGTGGTCCA
[3] Major Allele: TAGGGTAACG

Gene: Chromosome	Position	Imp.	Major	Minor	LD Group	Freq. High	Freq. Low	P value	refSNP ID	Genotyped
	76696535	intronic	A	G	19	0.29	0.38	0.13	rs3743074	
	76696594	intronic	T	G	19	0.29	0.38	0.13	rs3743073	
	76696612	intronic	G	A		0.01	<0.01	-	rs41280050	
	76698094	intronic	-	A	22	0.03	0.06	0.41	rs34844435	
	76698135	intronic	G	A		<0.01	0.01	-	rs56409843	
	76698236	synon	T	C	20	0.54	0.42	0.03	rs8040868	
	76698285	non	C	T	22	0.04	0.06	0.59	rs8192475	+
	76700428	flank	VNTR[4]						rs55787222	
**CHRNB4: Chr 15**										
	76703677	flank	G	C	19	0.29	0.39	0.08	rs2904130	
	76704371	UTR	C	T		0.02	0.01	0.67	rs55952530	
	76704454	UTR	G	A	19	0.27	0.38	0.05	rs1948	
	76708372	non	C	A		<0.01	<0.01	-	rs56258098	
	76708398	non	G	A		0.01	<0.01	-	rs56317523	+
	76708657	non	G	A		0.01	0.01	-	rs56235003	
	76708817	synon	G	A	23	0.01	0.01	-	rs3743072	
	76708862	synon	G	A		0.00	0.01	-	rs55859769	
	76709249	synon	G	A		0.05	0.04	1.00	rs55919125	
	76709284	non	T	C	23	<0.01	0.01	-	rs56218866	+
	76709288	synon	G	A		0.01	<0.01	-	rs55851389	
	76709295	non	C	T		0.01	0.02	0.62	rs56095004	+
	76710560	non	G	A	22	0.04	0.07	0.29	rs12914008	
	76710776	intronic	C	G		0.01	<0.01	-	rs55647022	
	76710900	intronic	T	G	21	0.16	0.22	0.23	rs28534575	
	76711042	intronic	C	T	20	0.48	0.28	0.001	rs17487223	
	76713781	intronic	C	G	21	0.17	0.22	0.43	rs12440014	
	76714644	intronic	T	G		0.01	0.01	-	rs12440298	
	76714827	intronic	T	C		<0.01	0.01	-	rs56011563	
	76715319	intronic	T	G		0.20	0.21	1.00	rs12441088	
**CHRNA4: Chr 20**										
	61446974	UTR	C	T	26	0.06	0.06	1.00	rs45476096	+
	61447364	UTR	T	C	26	0.06	0.06	1.00	rs41283006	
	61447445	UTR	C	T		<0.01	<0.01	-	rs55985535	
	61447459	UTR	C	T	26	0.06	0.06	1.00	rs41283008	
	61447493	UTR	G	C	26	0.06	0.06	1.00	rs41283010	
	61447553	UTR	C	T		0.01	0.01	-	rs55715203	

[4] Variable Nucleotide Repeat Frequencies: 4(GGCG) 0.510, 2(GGCG) 0.465, 5(GGCG) 0.025

Gene: Chromosome	Position	Imp.	Major	Minor	LD Group	Freq. High	Freq. Low	P value	refSNP ID	Genotyped
	61447587	UTR	C	T		0.01	0.01	-	rs56339657	
	61447666	UTR	G	A		0.00	0.01	-	rs56164905	
	61447762	UTR	G	T		0.02	0.04	0.72	rs45607838	
	61447779	UTR	A	G		0.08	0.10	0.84	rs45624232	
	61447817	UTR	G	A	26	0.06	0.06	1.00	rs45556643	
	61451397	intronic	G	A		<0.01	0.01	-	rs56160350	
	61451435	intronic	T	C	29	0.17	0.17	1.00	rs3827020	
	61451438	intronic	G	A		0.10	0.05	0.24	rs45442394	
	61451546	non	G	A	31	<0.01	0.01	-	rs56000199	
	61451548	synon	T	C	30	0.41	0.42	1.00	rs1044397	+
	61451578	synon	A	G	30	0.41	0.41	1.00	rs1044396	+
	61451759	non	C	T		<0.01	0.02	0.25	rs55855125	
	61451806	synon	G	A		<0.01	<0.01	-	rs45569837	
	61451855	non	G	A	31	<0.01	0.01	-	rs55915440	
	61451980	synon	G	A	27	0.05	0.05	1.00	rs2229960	
	61451998	synon	A	C	28	0.13	0.10	0.55	rs2229959	
	61452004	synon	C	G		<0.01	0.01	-	rs56142348	
	61452201	non	G	A		0.01	<0.01	-	rs56175056	
	61452529	synon	G	A	27	0.05	0.05	1.00	rs1044394	+
	61452568	synon	G	A	28	0.13	0.10	0.43	rs1044393	+
	61452846	intronic	G	A	26	0.06	0.06	1.00	rs56151028	
	61452940	intronic	C	T	28	0.14	0.11	0.70	rs13041103	
	61452956	intronic	C	T	25	0.12	0.07	0.26	rs55731226	
	61452958	intronic	T	C		0.04	0.02	0.50	rs55786941	
	61452967	intronic	C	-	27	0.03	0.04	0.72	rs55869461	
	61457989	intronic	G	-	25	0.27	0.08	0.13	rs56305048	
	61458015	intronic	C	T		0.08	0.00	0.49	rs56148915	
	61458016	intronic	G	A	25	0.21	0.08	0.42	rs56019702	
	61458019	intronic	A	G		<0.01	<0.01	-	rs55683444	
	61458266	intronic	C	T	25	0.41	0.46	0.81	rs2273502	+
	61461175	intronic	G	T		0.04	0.04	0.77	rs45539234	
	61461318	intronic	C	T	27	0.04	0.04	1.00	rs6090384	
	61461322	intronic	C	T	25	0.09	0.06	0.26	rs2273505	
	61461383	synon	G	A	25	0.09	0.06	0.26	rs2273506	
	61461585	intronic	G	A	29	0.21	0.18	0.65	rs3818204	+
	61461665	intronic	G	C		0.17	0.17	1.00	rs13044665	
