# Supplementary material for: A Candidate Gene Approach Identifies the CHRNA5-A3-B4 Region as a Risk Factor for Age-Dependent Nicotine Addiction
Source: PLoS Genet. 2008 Jul 11;4(7):e1000125. doi: 10.1371/journal.pgen.1000125 (PMC2442220; doi:10.1371/journal.pgen.1000125)
Supplement: Table S3 — Association of nicotinic receptor variants using dichotomized FTND Scores within age of onset of daily smoking in the combined UT and WI cohorts. (0.23 MB DOC) [file pgen.1000125.s003.doc]

**Table S3.** Survey of nicotinic receptor variants by association analyses using dichotomized FTND scores within age of onset of daily smoking in the combined UT and WI cohorts. Markers, locations, minor allele frequencies (MAF), allele test *P*-values (*P*-value^1^ = Fisher’s exact test, *P*-value^2^ = Cochran-Armitage trend test) are shown for age of onset of daily smoking classified as early onset (onset of daily smoking by age 16) and late onset (onset of daily smoking after age 16). Genotypes were filtered using thresholds for: minor allele frequency (<0.002), missing genotyping rate (<0.2) and Hardy-Weinberg equilibrium tests in case and controls (p < 1 x 10^-7^). “Imp” = functional implication of SNP, “UTR” = untranslated exon region, flank = flanking region, “synon” = synonymous codon change, “non” = nonsynonymous codon change. Genomic locations are base pair coordinates from NCBI Build 35.

|  |  |  |  |  |  | **Early onset of daily smoking** | | | | **Late onset of daily smoking** | | | |
| --- | --- | --- | --- | --- | --- | --- | --- | --- | --- | --- | --- | --- | --- |
| **Marker** | **Gene** | **Imp** | **Chr** | **Location** | **Min. allele** | **FTND =0-4 (n=106) MAF** | **FTND =6-10 (n=271) MAF** | ***P*-value^1^** | ***P*-value^2^** | **FTND =0-4 (n=183) MAF** | **FTND =6-10 (n=217) MAF** | ***P*-value^1^** | ***P*-value^2^** |
| rs2280781 | CHRNB2 | UTR | 1 | 151353541 | T | 0.080 | 0.081 | 0.95 | 0.95 | 0.086 | 0.078 | 0.69 | 0.68 |
| rs2072659 | CHRNB2 | UTR | 1 | 151361594 | G | 0.078 | 0.096 | 0.46 | 0.44 | 0.080 | 0.084 | 0.83 | 0.82 |
| rs2072660 | CHRNB2 | UTR | 1 | 151361794 | T | 0.197 | 0.262 | 0.064 | 0.06 | 0.271 | 0.227 | 0.15 | 0.13 |
| rs4292956 | CHRNB2 | UTR | 1 | 151362019 | T | 0.029 | 0.055 | 0.14 | 0.13 | 0.065 | 0.062 | 0.86 | 0.86 |
| rs4469116 | CHRNA9 | flank | 4 | 40178176 | T | 0.243 | 0.261 | 0.61 | 0.60 | 0.253 | 0.261 | 0.80 | 0.80 |
| rs10022491 | CHRNA9 | synon | 4 | 40178836 | T | 0.457 | 0.433 | 0.56 | 0.57 | 0.428 | 0.425 | 0.93 | 0.93 |
| rs6819385 | CHRNA9 | intron | 4 | 40180502 | G | 0.481 | 0.478 | 0.94 | 0.94 | 0.492 | 0.495 | 0.92 | 0.92 |
| rs4861065 | CHRNA9 | intron | 4 | 40185323 | C | 0.289 | 0.308 | 0.61 | 0.61 | 0.313 | 0.299 | 0.67 | 0.67 |
| rs10027533 | CHRNA9 | intron | 4 | 40185516 | C | 0.154 | 0.121 | 0.24 | 0.23 | 0.141 | 0.157 | 0.54 | 0.55 |
| rs10029313 | CHRNA9 | intron | 4 | 40191075 | T | 0.332 | 0.376 | 0.27 | 0.26 | 0.365 | 0.338 | 0.42 | 0.41 |
| rs7669882 | CHRNA9 | intron | 4 | 40191579 | A | 0.289 | 0.304 | 0.68 | 0.68 | 0.313 | 0.298 | 0.65 | 0.65 |
| rs10006948 | CHRNA9 | flank | 4 | 40199843 | G | 0.466 | 0.481 | 0.71 | 0.72 | 0.500 | 0.495 | 0.90 | 0.89 |
|  |  |  |  |  |  | **Early onset of daily smoking** | | | | **Late onset of daily smoking** | | | |
| **Marker** | **Gene** | **Imp** | **Chr** | **Location** | **Min. allele** | **FTND =0-4 (n=106) MAF** | **FTND =6-10 (n=271) MAF** | ***P*-value^1^** | ***P*-value^2^** | **FTND =0-4 (n=183) MAF** | **FTND =6-10 (n=217) MAF** | ***P*-value^1^** | ***P*-value^2^** |
| rs7812782 | CHRNA2 | flank | 8 | 27373254 | A | 0.204 | 0.136 | 0.021 | 0.019 | 0.127 | 0.131 | 0.87 | 0.87 |
| rs2280375 | CHRNA2 | flank | 8 | 27373904 | A | 0.188 | 0.128 | 0.040 | 0.034 | 0.105 | 0.135 | 0.21 | 0.21 |
| rs1560344 | CHRNA2 | flank | 8 | 27374342 | G | 0.414 | 0.462 | 0.23 | 0.23 | 0.492 | 0.479 | 0.72 | 0.73 |
| rs2292975 | CHRNA2 | UTR | 8 | 27375209 | A | 0.409 | 0.459 | 0.22 | 0.21 | 0.489 | 0.469 | 0.59 | 0.60 |
| rs735421 | CHRNA2 | intron | 8 | 27380739 | G | 0.264 | 0.320 | 0.14 | 0.16 | 0.331 | 0.380 | 0.15 | 0.15 |
| rs891398 | CHRNA2 | non | 8 | 27380761 | C | 0.476 | 0.472 | 0.92 | 0.92 | 0.458 | 0.529 | 0.049 | 0.049 |
| rs2565061 | CHRNA2 | synon | 8 | 27382044 | A | 0.188 | 0.128 | 0.040 | 0.032 | 0.111 | 0.129 | 0.45 | 0.45 |
| rs2741343 | CHRNA2 | intron | 8 | 27384271 | C | 0.481 | 0.483 | 0.95 | 0.95 | 0.463 | 0.529 | 0.069 | 0.069 |
| rs2472554 | CHRNA2 | intron | 8 | 27384428 | C | 0.264 | 0.305 | 0.28 | 0.29 | 0.315 | 0.282 | 0.32 | 0.33 |
| rs2472553 | CHRNA2 | non | 8 | 27386730 | T | 0.187 | 0.151 | 0.25 | 0.26 | 0.130 | 0.149 | 0.47 | 0.50 |
| rs2565045 | CHRNA2 | intron | 8 | 27386854 | G | 0.136 | 0.168 | 0.30 | 0.33 | 0.172 | 0.153 | 0.50 | 0.52 |
| rs7819756 | CHRNA2 | intron | 8 | 27387036 | C | 0.389 | 0.472 | 0.04 | 0.043 | 0.542 | 0.462 | 0.026 | 0.026 |
| rs2565067 | CHRNA2 | intron | 8 | 27391815 | T | 0.106 | 0.151 | 0.11 | 0.12 | 0.181 | 0.169 | 0.67 | 0.66 |
| rs2741337 | CHRNA2 | intron | 8 | 27373254 | T | 0.216 | 0.224 | 0.81 | 0.82 | 0.235 | 0.262 | 0.38 | 0.37 |
| rs4950 | CHRNB3A6 | UTR | 8 | 42671790 | C | 0.231 | 0.217 | 0.68 | 0.68 | 0.209 | 0.196 | 0.66 | 0.67 |
| rs13261190 | CHRNB3A6 | intron | 8 | 42697466 | G | 0.092 | 0.062 | 0.15 | 0.15 | 0.102 | 0.089 | 0.53 | 0.55 |
| rs16891561 | CHRNB3A6 | intron | 8 | 42698896 | T | 0.192 | 0.181 | 0.72 | 0.73 | 0.178 | 0.173 | 0.86 | 0.87 |
| rs4953 | CHRNB3A6 | synon | 8 | 42706816 | C | 0.048 | 0.045 | 0.86 | 0.86 | 0.040 | 0.031 | 0.52 | 0.51 |
| rs7012713 | CHRNB3A6 | flank | 8 | 42711460 | T | 0.024 | 0.022 | 0.87 | 0.87 | 0.033 | 0.026 | 0.55 | 0.55 |
| rs7017612 | CHRNB3A6 | flank | 8 | 42718402 | C | 0.216 | 0.197 | 0.56 | 0.55 | 0.196 | 0.189 | 0.81 | 0.81 |
| rs9298629 | CHRNB3A6 | flank | 8 | 42725343 | T | 0.202 | 0.200 | 0.94 | 0.95 | 0.192 | 0.185 | 0.78 | 0.78 |
| rs892413 | CHRNB3A6 | intron | 8 | 42733535 | A | 0.202 | 0.190 | 0.71 | 0.72 | 0.189 | 0.179 | 0.70 | 0.70 |
| rs17621710 | CHRNB3A6 | intron | 8 | 42734717 | T | 0.106 | 0.120 | 0.58 | 0.56 | 0.128 | 0.121 | 0.79 | 0.78 |
| rs16891604 | CHRNB3A6 | intron | 8 | 42737870 | A | 0.058 | 0.050 | 0.66 | 0.67 | 0.060 | 0.063 | 0.88 | 0.88 |
| rs1072003 | CHRNB3A6 | intron | 8 | 42739158 | G | 0.197 | 0.173 | 0.44 | 0.46 | 0.170 | 0.182 | 0.65 | 0.65 |
| rs2741862 | CHRNA10 | intron | 11 | 3644561 | C | 0.248 | 0.255 | 0.84 | 0.84 | 0.242 | 0.239 | 0.94 | 0.94 |
| rs2741868 | CHRNA10 | intron | 11 | 3646759 | T | 0.317 | 0.349 | 0.41 | 0.41 | 0.302 | 0.325 | 0.50 | 0.49 |
| rs2672213 | CHRNA10 | intron | 11 | 3647993 | T | 0.185 | 0.159 | 0.41 | 0.39 | 0.170 | 0.150 | 0.43 | 0.43 |
| rs2231532 | CHRNA10 | flank | 11 | 3649696 | A | 0.423 | 0.439 | 0.70 | 0.70 | 0.395 | 0.435 | 0.26 | 0.24 |
|  |  |  |  |  |  | **Early onset of daily smoking** | | | | **Late onset of daily smoking** | | | |
| **Marker** | **Gene** | **Imp** | **Chr** | **Location** | **Min. allele** | **FTND =0-4 (n=106) MAF** | **FTND =6-10 (n=271) MAF** | ***P*-value^1^** | ***P*-value^2^** | **FTND =0-4 (n=183) MAF** | **FTND =6-10 (n=217) MAF** | ***P*-value^1^** | ***P*-value^2^** |
| rs2231529 | CHRNA10 | flank | 11 | 3649829 | T | 0.115 | 0.093 | 0.37 | 0.36 | 0.096 | 0.101 | 0.84 | 0.84 |
| rs4575303 | CHRNA10 | flank | 11 | 3663022 | G | 0.221 | 0.191 | 0.36 | 0.35 | 0.174 | 0.203 | 0.30 | 0.28 |
| rs883473 | CHRNA7 | intron | 15 | 30112968 | T | 0.350 | 0.292 | 0.13 | 0.12 | 0.296 | 0.258 | 0.24 | 0.23 |
| rs6494182 | CHRNA7 | intron | 15 | 30132987 | A | 0.074 | 0.046 | 0.15 | 0.14 | 0.052 | 0.059 | 0.71 | 0.71 |
| rs8037484 | CHRNA7 | intron | 15 | 30134166 | A | 0.308 | 0.229 | 0.03 | 0.02 | 0.240 | 0.210 | 0.31 | 0.33 |
| rs7179733 | CHRNA7 | intron | 15 | 30160985 | C | 0.490 | 0.422 | 0.09 | 0.08 | 0.445 | 0.430 | 0.68 | 0.67 |
| rs1514250 | CHRNA7 | intron | 15 | 30164998 | G | 0.113 | 0.095 | 0.48 | 0.48 | 0.088 | 0.101 | 0.53 | 0.53 |
| rs7175581 | CHRNA7 | intron | 15 | 30172759 | A | 0.471 | 0.422 | 0.22 | 0.23 | 0.445 | 0.423 | 0.53 | 0.53 |
| rs8036104 | CHRNA7 | intron | 15 | 30176654 | C | 0.144 | 0.184 | 0.20 | 0.22 | 0.181 | 0.180 | 0.96 | 0.96 |
| rs12438848 | CHRNA7 | intron | 15 | 30183663 | T | 0.456 | 0.406 | 0.22 | 0.24 | 0.379 | 0.414 | 0.32 | 0.32 |
| rs10438342 | CHRNA7 | intron | 15 | 30189338 | A | 0.341 | 0.353 | 0.76 | 0.76 | 0.321 | 0.340 | 0.57 | 0.58 |
| rs12915265 | CHRNA7 | intron | 15 | 30196358 | C | 0.235 | 0.221 | 0.68 | 0.69 | 0.192 | 0.220 | 0.34 | 0.34 |
| rs2221223 | CHRNA7 | intron | 15 | 30198685 | C | 0.120 | 0.149 | 0.32 | 0.33 | 0.137 | 0.136 | 0.94 | 0.94 |
| rs904951 | CHRNA7 | intron | 15 | 30205330 | G | 0.447 | 0.519 | 0.078 | 0.077 | 0.511 | 0.455 | 0.12 | 0.12 |
| rs7178176 | CHRNA7 | intron | 15 | 30231105 | T | 0.320 | 0.331 | 0.79 | 0.78 | 0.302 | 0.245 | 0.073 | 0.071 |
| rs2337980 | CHRNA7 | intron | 15 | 30231488 | T | 0.442 | 0.506 | 0.12 | 0.12 | 0.442 | 0.428 | 0.68 | 0.68 |
| rs17486278 | CHRNA5A3B4 | intron | 15 | 76654537 | C | 0.279 | 0.417 | 0.0005 | 0.00037 | 0.403 | 0.395 | 0.81 | 0.81 |
| rs680244 | CHRNA5A3B4 | intron | 15 | 76658343 | A | 0.457 | 0.416 | 0.32 | 0.30 | 0.409 | 0.411 | 0.96 | 0.96 |
| rs569207 | CHRNA5A3B4 | intron | 15 | 76660174 | A | 0.260 | 0.167 | 0.0042 | 0.0028 | 0.191 | 0.203 | 0.68 | 0.68 |
| rs555018 | CHRNA5A3B4 | intron | 15 | 76666297 | C | 0.462 | 0.415 | 0.25 | 0.24 | 0.407 | 0.400 | 0.85 | 0.85 |
| rs2229961 | CHRNA5A3B4 | non | 15 | 76667807 | A | 0.021 | 0.030 | 0.49 | 0.49 | 0.015 | 0.029 | 0.18 | 0.18 |
| rs12903575 | CHRNA5A3B4 | intron | 15 | 76668142 | A | 0.053 | 0.053 | 0.97 | 0.97 | 0.034 | 0.024 | 0.40 | 0.39 |
| rs16969968 | CHRNA5A3B4 | non | 15 | 76669980 | A | 0.288 | 0.417 | 0.0009 | 0.00078 | 0.399 | 0.392 | 0.84 | 0.84 |
| rs578776 | CHRNA5A3B4 | UTR | 15 | 76675455 | T | 0.317 | 0.218 | 0.0048 | 0.0033 | 0.218 | 0.223 | 0.87 | 0.87 |
| rs1051730 | CHRNA5A3B4 | synon | 15 | 76681394 | T | 0.284 | 0.415 | 0.0009 | 0.00071 | 0.398 | 0.401 | 0.93 | 0.93 |
| rs2869546 | CHRNA5A3B4 | intron | 15 | 76694400 | C | 0.394 | 0.374 | 0.61 | 0.61 | 0.384 | 0.374 | 0.77 | 0.77 |
| rs7177514 | CHRNA5A3B4 | intron | 15 | 76694461 | G | 0.341 | 0.235 | 0.0032 | 0.0064 | 0.229 | 0.245 | 0.59 | 0.62 |
| rs12443170 | CHRNA5A3B4 | intron | 15 | 76694791 | A | 0.149 | 0.096 | 0.038 | 0.037 | 0.111 | 0.148 | 0.13 | 0.13 |
| rs8192475 | CHRNA5A3B4 | non | 15 | 76698285 | A | 0.054 | 0.049 | 0.77 | 0.76 | 0.040 | 0.031 | 0.50 | 0.49 |
|  |  |  |  |  |  | **Early onset of daily smoking** | | | | **Late onset of daily smoking** | | | |
| **Marker** | **Gene** | **Imp** | **Chr** | **Location** | **Min. allele** | **FTND =0-4 (n=106) MAF** | **FTND =6-10 (n=271) MAF** | ***P*-value^1^** | ***P*-value^2^** | **FTND =0-4 (n=183) MAF** | **FTND =6-10 (n=217) MAF** | ***P*-value^1^** | ***P*-value^2^** |
| rs56317523 | CHRNA5A3B4 | non | 15 | 76708398 | T | 0.005 | 0.004 | 0.83 | 0.83 | 0.006 | 0.002 | 0.45 | 0.45 |
| rs56218866 | CHRNA5A3B4 | non | 15 | 76709284 | G | 0.010 | 0.010 | 0.98 | 0.98 | 0.012 | 0.000 | 0.027 | 0.027 |
| rs56095004 | CHRNA5A3B4 | non | 15 | 76709295 | A | 0.005 | 0.008 | 0.69 | 0.68 | 0.015 | 0.007 | 0.32 | 0.32 |
| rs11636605 | CHRNA5A3B4 | intron | 15 | 76715933 | A | 0.212 | 0.156 | 0.072 | 0.072 | 0.159 | 0.182 | 0.40 | 0.40 |
| rs11633223 | CHRNA5A3B4 | flank | 15 | 76722531 | C | 0.414 | 0.378 | 0.37 | 0.36 | 0.384 | 0.376 | 0.81 | 0.81 |
| rs3971872 | CHRNA5A3B4 | flank | 15 | 76729090 | T | 0.096 | 0.092 | 0.86 | 0.86 | 0.068 | 0.079 | 0.57 | 0.57 |
| gs48841 | CHRNA4 | UTR | 20 | 61446974 | A | 0.058 | 0.049 | 0.61 | 0.61 | 0.055 | 0.068 | 0.45 | 0.43 |
| rs2236196 | CHRNA4 | UTR | 20 | 61448000 | G | 0.301 | 0.277 | 0.52 | 0.52 | 0.265 | 0.282 | 0.60 | 0.58 |
| rs3787138 | CHRNA4 | intron | 20 | 61449668 | G | 0.130 | 0.154 | 0.40 | 0.43 | 0.119 | 0.148 | 0.24 | 0.22 |
| rs1044397 | CHRNA4 | synon | 20 | 61451548 | G | 0.495 | 0.468 | 0.50 | 0.49 | 0.463 | 0.442 | 0.56 | 0.55 |
| rs1044396 | CHRNA4 | synon | 20 | 61451578 | G | - | - | - | - | - | - | - | - |
| rs1044394 | CHRNA4 | synon | 20 | 61452529 | T | 0.082 | 0.077 | 0.83 | 0.83 | 0.054 | 0.069 | 0.39 | 0.37 |
| rs1044393 | CHRNA4 | synon | 20 | 61452568 | T | 0.130 | 0.151 | 0.46 | 0.47 | 0.113 | 0.141 | 0.24 | 0.22 |
| rs2273502 | CHRNA4 | intron | 20 | 61458266 | T | 0.077 | 0.085 | 0.73 | 0.73 | 0.071 | 0.081 | 0.59 | 0.59 |
| rs2273504 | CHRNA4 | intron | 20 | 61458505 | A | 0.186 | 0.161 | 0.41 | 0.42 | 0.185 | 0.139 | 0.075 | 0.06 |
| rs6010918 | CHRNA4 | intron | 20 | 61459945 | A | 0.053 | 0.066 | 0.51 | 0.51 | 0.045 | 0.057 | 0.45 | 0.43 |
| rs3818204 | CHRNA4 | intron | 20 | 61461585 | A | 0.160 | 0.145 | 0.59 | 0.60 | 0.160 | 0.114 | 0.066 | 0.061 |
