# Supplementary material for: A Candidate Gene Approach Identifies the CHRNA5-A3-B4 Region as a Risk Factor for Age-Dependent Nicotine Addiction
Source: PLoS Genet. 2008 Jul 11;4(7):e1000125. doi: 10.1371/journal.pgen.1000125 (PMC2442220; doi:10.1371/journal.pgen.1000125)
Supplement: Table S4 — Results of logistic regression analyses in which Haplotype C was the reference condition and two alternative criteria for low and high nicotine dependence were used (0–4 vs. 5–10 and 0–5 vs. 6–10). (0.01 MB PDF) [file pgen.1000125.s004.pdf]

Table S4. Results of logistic regression analyses in which Haplotype C was the reference condition and two alternative criteria for low and high nicotine dependence were used (0-4 vs. 5-10 and 0-5 vs. 6-10).

**FTND Split**	**Hap**	**Interaction**		**Early Onset**[1]		**Late Onset**[2]	
		P	**OR**	***P***	**OR**	***P***	**OR**
**0-4 vs. 5-10**	**H_A**	0.03	1.54 (1.06-2.25)	0.0001	1.77 (1.32-2.38)	0.26	1.15 (0.90-1.46)
	**H_B**	0.32	1.21 (0.83-1.75)	0.08	1.29 (0.97-1.71)	0.59	1.07 (0.84-1.36)
**0-5 vs. 6-10**	**H_A**	0.04	1.43 (1.02-2.01)	0.00009	1.65 (1.28-2.12)	0.22	1.16 (0.92-1.45)
	**H_B**	0.10	1.33 (0.95-1.87)	0.04	1.31 (1.02-1.68)	0.91	1.01 (0.81-1.28)

"Hap" = haplotype, "Interaction" = interaction between haplotype and early vs. late onset of daily smoking, "OR" = odds ratio (95% confidence intervals in parentheses).
[1] "Early Onset" = age of onset of daily smoking by 16.
[2] "Late Onset" = age of onset of daily smoking after 16.
