# Supplementary material for: A Candidate Gene Approach Identifies the CHRNA5-A3-B4 Region as a Risk Factor for Age-Dependent Nicotine Addiction
Source: PLoS Genet. 2008 Jul 11;4(7):e1000125. doi: 10.1371/journal.pgen.1000125 (PMC2442220; doi:10.1371/journal.pgen.1000125)
Supplement: Table S5 — Frequency of individual CHRNA5-A3 SNPs in the UT-WI-LHS cohorts according to individual FTND items. (0.12 MB DOC) [file pgen.1000125.s005.doc]

**Table S5**. Frequency of individual *CHRNA5-A3* SNPs in 2,827 subjects from the UT-WI-LHS cohorts according to individual FTND items.

| **FTND Item (score)** | **Early Onset^1^** | | | | | | **Late Onset^2^** | | | | | | **Combined** | | | | | |
| --- | --- | --- | --- | --- | --- | --- | --- | --- | --- | --- | --- | --- | --- | --- | --- | --- | --- | --- |
|  | **n** | **rs680244** | **rs569207** | **rs16969968** | **rs578776** | **rs1051730** | **n** | **rs680244** | **rs569207** | **rs16969968** | **rs578776** | **rs1051730** | **n** | **rs680244** | **rs569207** | **rs16969968** | **rs578776** | **rs1051730** |
| **1. How soon after you wake up do you smoke your first cigarette? (max. 3)** | | | | | | | | |  |  |  |  |  |  |  |  |  |  |
| > 60 min (0) | 60 | 0.425 | 0.233 | 0.342 | 0.317 | 0.342 | 125 | 0.436 | 0.200 | 0.364 | 0.236 | 0.364 | 185 | 0.432 | 0.211 | 0.357 | 0.262 | 0.357 |
| 31-60 min (1) | 131 | 0.439 | 0.237 | 0.324 | 0.294 | 0.324 | 225 | 0.460 | 0.180 | 0.360 | 0.227 | 0.360 | 356 | 0.452 | 0.201 | 0.347 | 0.251 | 0.347 |
| 6-30 min (2) | 482 | 0.402 | 0.193 | 0.405 | 0.248 | 0.405 | 626 | 0.394 | 0.194 | 0.412 | 0.238 | 0.412 | 1108 | 0.398 | 0.194 | 0.409 | 0.242 | 0.409 |
| < 6 min (3) | 614 | 0.424 | 0.191 | 0.384 | 0.242 | 0.384 | 564 | 0.418 | 0.181 | 0.402 | 0.213 | 0.402 | 1178 | 0.421 | 0.186 | 0.393 | 0.228 | 0.393 |
| **2. Do you find it difficult to refrain from smoking in places where it is forbidden?** | | | | | | | | | |  |  |  |  |  |  |  |  |  |
| Yes (1) | 336 | 0.417 | 0.204 | 0.379 | 0.249 | 0.379 | 308 | 0.424 | 0.187 | 0.390 | 0.214 | 0.390 | 644 | 0.420 | 0.196 | 0.384 | 0.232 | 0.384 |
| No (0) | 951 | 0.418 | 0.197 | 0.385 | 0.254 | 0.385 | 1232 | 0.414 | 0.188 | 0.399 | 0.230 | 0.399 | 2183 | 0.415 | 0.192 | 0.393 | 0.241 | 0.393 |
| **3. Which cigarette would you hate most to give up, first one in the morning?** | | | | | | | | | |  |  |  |  |  |  |  |  |  |
| First (1) | 714 | 0.403 | 0.200 | 0.398 | 0.251 | 0.398 | 826 | 0.413 | 0.185 | 0.402 | 0.223 | 0.402 | 1540 | 0.408 | 0.192 | 0.400 | 0.236 | 0.400 |
| Any other (0) | 573 | 0.436 | 0.197 | 0.366 | 0.256 | 0.366 | 714 | 0.419 | 0.190 | 0.391 | 0.232 | 0.391 | 1287 | 0.427 | 0.193 | 0.380 | 0.242 | 0.380 |
| **4. How many cigarettes per day did/do you smoke?** | | | | | |  |  |  |  |  |  |  |  |  |  |  |  |  |
| < 11 (0) | 71 | 0.423 | 0.275 | 0.303 | 0.310 | 0.303 | 146 | 0.432 | 0.182 | 0.387 | 0.226 | 0.387 | 217 | 0.429 | 0.212 | 0.359 | 0.253 | 0.359 |
| 11-20 (1) | 378 | 0.425 | 0.216 | 0.360 | 0.275 | 0.360 | 537 | 0.428 | 0.203 | 0.369 | 0.237 | 0.369 | 915 | 0.427 | 0.208 | 0.365 | 0.253 | 0.365 |
| 21-30 (2) | 407 | 0.429 | 0.203 | 0.369 | 0.254 | 0.369 | 404 | 0.418 | 0.173 | 0.408 | 0.218 | 0.408 | 811 | 0.424 | 0.188 | 0.388 | 0.236 | 0.388 |
| >30 (3) | 431 | 0.400 | 0.167 | 0.433 | 0.223 | 0.433 | 453 | 0.393 | 0.184 | 0.423 | 0.223 | 0.423 | 884 | 0.396 | 0.176 | 0.428 | 0.223 | 0.428 |
| **5. Do you smoke more frequently during the first hours after waking than during the rest of the day?** | | | | | | | | | | | | |  |  |  |  |  |  |
| Yes (1) | 471 | 0.414 | 0.183 | 0.403 | 0.230 | 0.403 | 499 | 0.405 | 0.187 | 0.408 | 0.220 | 0.408 | 970 | 0.409 | 0.185 | 0.406 | 0.225 | 0.406 |
| No (0) | 509 | 0.420 | 0.213 | 0.366 | 0.263 | 0.366 | 656 | 0.423 | 0.190 | 0.387 | 0.231 | 0.387 | 1165 | 0.422 | 0.197 | 0.378 | 0.245 | 0.378 |
| **6. Do you smoke if you are so ill that you are in bed most of the day?** | | | | | | | | |  |  |  |  |  |  |  |  |  |  |
| Yes (1) | 770 | 0.408 | 0.192 | 0.401 | 0.240 | 0.401 | 724 | 0.408 | 0.189 | 0.403 | 0.227 | 0.403 | 1494 | 0.408 | 0.190 | 0.402 | 0.233 | 0.402 |
| No (0) | 517 | 0.432 | 0.209 | 0.359 | 0.273 | 0.359 | 816 | 0.422 | 0.187 | 0.391 | 0.227 | 0.391 | 1333 | 0.426 | 0.195 | 0.378 | 0.245 | 0.378 |

The minor alleles are from the chr. 15 (+) strand: rs680244 (T), rs569207 (T), rs16969968 (A), rs578776 (A), and rs1051730 (A). ‘n’ = number of subjects responding to questionnaire item.

^1^ “Early Onset” = age of onset of daily smoking by 16. ^2^ “Late Onset” = age of onset after 16.
