# Supplementary material for: A Candidate Gene Approach Identifies the CHRNA5-A3-B4 Region as a Risk Factor for Age-Dependent Nicotine Addiction
Source: PLoS Genet. 2008 Jul 11;4(7):e1000125. doi: 10.1371/journal.pgen.1000125 (PMC2442220; doi:10.1371/journal.pgen.1000125)
Supplement: Table S6 — Frequency of Haplotypes A, B and C in the UT-WI-LHS cohorts according to individual FTND items. (0.01 MB PDF) [file pgen.1000125.s006.pdf]

Table S6. Frequency of Haplotypes A, B and C in 2,827 subjects from the UT-WI-LHS cohorts according to individual FTND items.

FTND Item	Early Onset[1] Haplotype Frequency				Late Onset[2] Haplotype Frequency			
	n	A	B	C	n	A	B	C
**1. How soon after you wake up do you smoke your first cigarette? (max. 3)**								
> 60 min (0)	60	0.342	0.342	0.233	125	0.364	0.400	0.200
31-60 min (1)	131	0.324	0.378	0.237	225	0.360	0.413	0.180
6-30 min (2)	482	0.404	0.346	0.193	626	0.412	0.350	0.194
< 6 min (3)	614	0.384	0.374	0.191	564	0.402	0.383	0.181
**2. Do you find it difficult to refrain from smoking in places where it is forbidden?**								
Yes (1)	336	0.379	0.371	0.204	308	0.390	0.394	0.187
No (0)	951	0.385	0.360	0.197	1232	0.399	0.371	0.188
**3. Which cigarette would you hate most to give up, first one in the morning?**								
First (1)	714	0.398	0.350	0.200	826	0.402	0.374	0.185
Any other (0)	573	0.366	0.378	0.197	714	0.391	0.377	0.190
**4. How many cigarettes per day did/do you smoke?**								
< 11 (0)	71	0.303	0.380	0.275	146	0.387	0.387	0.182
11-20 (1)	378	0.360	0.365	0.216	537	0.369	0.392	0.203
21-30 (2)	407	0.369	0.377	0.203	404	0.408	0.374	0.173
>30 (3)	431	0.433	0.343	0.167	453	0.423	0.353	0.184
**5. Do you smoke more frequently during the first hours after waking than during the rest of the day?**								
Yes (1)	471	0.403	0.365	0.183	499	0.408	0.372	0.187
No (0)	509	0.366	0.369	0.213	656	0.387	0.380	0.190
**6. Do you smoke if you are so ill that you are in bed most of the day?**								
Yes (1)	770	0.401	0.359	0.192	724	0.403	0.368	0.189
No (0)	517	0.359	0.368	0.209	816	0.391	0.382	0.187

The haplotype-tagging SNPs are rs680244, rs569207, rs16969968, rs578776, and rs1051730, and tag sequences are from the chr. 15 (+) strand; Haplotype A tag = CCAGA, Haplotype B tag = TCGGG, Haplotype C tag = CTGAG. ‘n’ = number of subjects responding to questionnaire item.

[1] “Early Onset” = age of onset of daily smoking by 16.
[2] “Late Onset” = age of onset of daily smoking after 16.
