# Supplementary material for: A Candidate Gene Approach Identifies the CHRNA5-A3-B4 Region as a Risk Factor for Age-Dependent Nicotine Addiction
Source: PLoS Genet. 2008 Jul 11;4(7):e1000125. doi: 10.1371/journal.pgen.1000125 (PMC2442220; doi:10.1371/journal.pgen.1000125)
Supplement: Table S7 — rs1051730 genotype and FTND item 4 comparison between combined early and late onset UT-WI-LHS smokers versus Icelandic smokers. (0.01 MB PDF) [file pgen.1000125.s007.pdf]

Table S7. rs1051730 genotype and FTND item 4 comparison between combined early and late onset UT-WI-LHS smokers versus Icelandic smokers.

Study	FTND Item 4	Genotype of rs1051730			Total n (frequency)	Frequency of T allele
		CC	CT	TT		
UT-WI-LHS[a]						
	Cigarettes per day (SQ level)					
	1 to 10 (0)	94	90	33	217 (0.077)	0.359
	11 to 20 (1)	362	438	115	915 (0.324)	0.365
	21 to 30 (2)	309	374	128	811 (0.287)	0.388
	31 and more (3)	288	436	160	884 (0.313)	0.428
	All levels	1,053	1,338	436	2,827 (1.000)	
	(frequency)	(0.372)	(0.473)	(0.154)		0.391
	Mean SQ level	1.75	1.86	1.95		
Icelandic Smokers[b]						
	Cigarettes per day (SQ level)					
	1 to 10 (0)	1,743	1,558	326	3,627 (0.260)	0.305
	11 to 20 (1)	2,727	2,865	810	6,402 (0.459)	0.350
	21 to 30 (2)	1,145	1,416	427	2,988 (0.214)	0.380
	31 and more (3)	341	448	139	928 (0.067)	0.391
	All levels	5,956	6,287	1,702	13,945 (1.000)	
	(frequency)	(0.427)	(0.451)	(0.122)		0.347
	Mean SQ level	1.01	1.12	1.22		

[a] data from this study, early and late onset combined, rs1051730 allele T is equivalent to Haplotype A in this population
[b] data from Table 1, Thorgeirsson et al., Nature April 2008, 452: 638-641., GG and GT genotypes from that table have been recoded to CC and CT to be consistent with our data and the NCBI Reference SNP Cluster Report for rs1051730.
